# Tyrosine kinase SRC-induced YAP1-KLF5 module regulates cancer stemness and metastasis in triple-negative breast cancer

**DOI:** 10.1007/s00018-023-04688-w

**Published:** 2023-01-12

**Authors:** Hailin Zou, Juan Luo, Yibo Guo, Tongyu Tong, Yuchen Liu, Yun Chen, Yunjun Xiao, Liping Ye, Chengming Zhu, Liang Deng, Bo Wang, Yihang Pan, Peng Li

**Affiliations:** 1 Scientific Research Center, The Seventh Affiliated Hospital of Sun Yat-Sen University, No. 628 Zhenyuan Road, Shenzhen, 518107 Guangdong People’s Republic of China; 2 Department of Urology, Pelvic Floor Disorders Center, The Seventh Affiliated Hospital of Sun Yat-Sen University, No. 628 Zhenyuan Road, Shenzhen, 518107 Guangdong People’s Republic of China; 3 Department of General Surgery, The Seventh Affiliated Hospital of Sun Yat-Sen University, No. 628 Zhenyuan Road, Shenzhen, 518107 Guangdong People’s Republic of China; 4 Department of Oncology, The Seventh Affiliated Hospital of Sun Yat-Sen University, No. 628 Zhenyuan Road, Shenzhen, 518107 Guangdong People’s Republic of China; 5 Guangdong Provincial Key Laboratory of Digestive Cancer Research, The Seventh Affiliated Hospital of Sun Yat-Sen University, No. 628 Zhenyuan Road, Shenzhen, 518107 Guangdong People’s Republic of China

**Keywords:** Triple-negative breast cancer, Cancer stem cell, SRC kinase, YAP1-KLF5 module

## Abstract

SRC is the first identified oncogene, and its aberrant activation has been implicated as a driving event in tumor initiation and progression. However, its role in cancer stemness regulation and the underlying regulatory mechanism are still elusive. Here, we identified a YAP1 tyrosine phosphorylation-dependent YAP1-KLF5 oncogenic module, as the key downstream mediator of SRC kinase regulating cancer stemness and metastasis in triple-negative breast cancer (TNBC). SRC was overexpressed in TNBC patient tissues and its expression level was highly correlated with the tumor malignancy. SRC activation induced, while inhibition of SRC kinase reduced the cancer stemness, tumor cell growth and metastasis in vitro and in vivo. Transcriptomic and proteomic analysis revealed that SRC-mediated YAP1 tyrosine phosphorylation induced its interaction with Kruppel-like factor 5 (KLF5) to form a YAP1/TEAD-KLF5 complex in TNBC cells. YAP1-KLF5 association further promoted TEAD-mediated transcriptional program independently of canonical Hippo kinases, which eventually gave rise to the enhanced cancer stemness and metastasis. Disruption of YAP1-KLF5 module in TNBC cells dramatically attenuated the SRC-induced cancer stemness and metastasis in vitro and in vivo. Accordingly, co-upregulations of SRC and YAP1-KLF5 module in TNBC tissues were significantly positively correlated with the tumor malignance. Altogether, our work presents a novel tyrosine phosphorylation-dependent YAP1-KLF5 oncogenic module governing SRC-induced cancer stemness and metastasis in TNBC. Therefore, targeting YAP1/KLF5-mediated transcription may provide a promising strategy for TNBC treatment with SRC aberrantly activation.

**Supplementary Information:**

The online version contains supplementary material available at 10.1007/s00018-023-04688-w.

## Introduction

BC is the leading cause of cancer-related deaths in women all around the world [1]. Among which, TNBC is the most aggressive BC subtype [2]. Due to the tumor heterogeneity of TNBC and lack of expressions of ER/PR and HER2, targeted therapies are very limited for this subgroup of BC patients. Despite these patients responding to conventional chemotherapy, most of the patients commonly develop chemo-resistance [3, 4]. In addition, the recently-reported immunotherapy by targeting PD-1/PD-L1 module, produced very limited improvement in overall survival of TNBC patients [5]. Thus, finding new therapeutic strategies is very urgent for curing TNBC. Accumulating studies have revealed that BCSCs primarily exist in TNBCs and these cell populations are positively correlated with the ‘triple-negative’ state and unfavorable prognosis of BC patients [6, 7]. Targeting BCSC regulation thus serves as a very promising strategy for TNBC treatment.

*c-SRC* is the first identified proto-oncogene, and it encodes a non-receptor protein-tyrosine kinase [8, 9]. This kinase is usually activated by the receptor tyrosine kinases, such as EGFR and PDGFR, and then transduces the extracellular stimuli into intracellular signals [10]. Structurally, SRC protein consists of four SRC-homology (SH1–SH4) domains and a C-terminal tail regulatory region. In addition, two tyrosine sites, including Tyr416 (an activating autophosphorylation site) and Tyr527 (an inhibitory phosphorylation site), have been proved to be essential for regulating SRC kinase activity [11, 12]. Specifically, SRC is inactive when Tyr527 residue is phosphorylated, owing that phosphorylated Tyr527 could bind to the SH2 domain and protect the catalytic pocket of Tyr416 in the kinase domain from inappropriate phosphorylation [13]. The pro-mitogenic growth factor treatments, such as EGF and PDGF, will induce the dephosphorylation of Tyr527, unlocking the catalytic pocket of Tyr416 and consequential de-inhibition of SRC kinase [14, 15]. Aberrant overexpression or activation of SRC kinase was frequently identified in various human malignant cancers, including BCs [16, 17]. Furthermore, SRC activation has been discovered to involve multiple processes underlying tumor development and progression, including cell proliferation, migration, invasion, and metastasis [10, 18]. Meanwhile, lots of the downstream components have been identified to be responsible for SRC kinase-mediated functions in different subtypes of BCs, including FAK, SGK1, LATS and so on [19–21].

Using the TNBC mouse model, a recent identification of the selective SRC family kinase (SFK) inhibitor eCF506 has shown a very high antitumor efficiency against both primary tumors and metastatic site tumors [22]. In TNBC patients, SRC pathway has also been identified to be one of the most commonly upregulated pathways [23, 24]. SRC kinase inhibitor Dasatinib alone, particularly combining with cisplatin has been shown great potential in treating metastatic TNBC [23, 25, 26]. Moreover, emerging preclinical evidence has demonstrated that TNBC cells showed higher sensitivity to the c-SRC inhibitor than do other cancer subgroups by targeting BCSCs [27–30], further highlighting the potential significance of targeting BCSCs through inhibiting SRC activity in TNBC treatment. However, the role of SRC kinase in cancer stemness regulation and the underlying regulatory mechanisms are still obscured.

Previous studies have reported that YAP1 could be phosphorylated at tyrosine 357 (YAP1 isoform with 454aa) by SFKs, including ABL, SRC and YES, which participated in DNA damage response, inflammation and tumorigenesis in various contexts. In the current study, we identify the YAP1-KLF5 oncogenic module as the key downstream target of SRC kinase to regulate the cancer stemness, cell proliferation and metastasis in TNBC cells, which is independent of canonical Hippo kinases. The SRC gain- and loss-of-function (GOF and LOF) assays demonstrated that SRC kinase regulated the cancer stemness and metastasis of TNBC cells in vitro and in vivo. Disruption of YAP1-KLF5 attenuated SRC activation-induced cancer stemness and metastasis. Furthermore, co-upregulations of SRC and YAP1-KLF5 module in TNBC tissues were positively correlated with the tumor malignance. Collectively, all our findings unraveled a novel molecular mechanism by which the TNBC stemness and aggressive behaviors can be developed through the SRC-YAP1/KLF5 signaling axis, and independently of the canonical Hippo kinases. Based on our findings, we proposed that targeting the YAP1/KLF5 module may represent a rational therapeutic strategy for SRC aberrantly activated TNBCs.

## Materials and methods

### Cell lines and DNA plasmids

Human MCF10A cell, 293 T and the human BC cell lines used in this study, including MDA-MB-231 and CAL51 were purchased from American Type Culture Collection (ATCC). MCF10A cells were grown in DMEM/F12 (Gibco), provided with 5% horse serum (Solarbio), 10 μg/mL insulin (MCE), 20 ng/ml epidermal growth factor (EGF) (MCE), 250 ng/ml Hydrocortisone (MCE), 100 ng/ml Cholera toxin (MCE) and 100 U/mL penicillin/streptomycin (Gibco). 293 T cell and all human BC cells were grown in DMEM (High glucose) (Gibco) supplemented with 10% fetal bovine serum (FBS) (Hyclone), 2 mmol/L L-glutamine (Gibco) and 100 U/mL penicillin/streptomycin (Gibco). The gene overexpressing plasmids (pUbi-MCS-3xFlag) and knockdown plasmids (pLKO.1-Puro) used for subcloning human *SRC, CA-SRC(Y530F), KLF5, YAP1* or *YAP1S127A* were purchased from GeneChem and TranSheepBio company respectively. Cell transfection, lentiviral production and infection assays were performed as previously described [31].

### Reverse-transcription, real-time PCR and western blot

All these procedures were performed as previously described [31]. The oligo sequences and the antibody information used in this study were listed in the Supplementary table 1 and table 2 respectively.

### Co-IP, luciferase assay and flow cytometry analysis

#### Co-IP

Flag-IP was performed as previously described using the Anti-Flag M2 magnetic beads (Sigma) according to the instructions [32]. The YAP1 and KLF5 antibody reciprocal Co-IP assay was performed using the protein A/G magnetic beads (MCE) according to the product instructions. In brief, the human cells, including 293 T, MCF10A and all BC cells were lysed first in 50 mM Tris–HCl (pH = 7.5), 100 mM NaCl, 1% Triton X-100, 0.1 mM EDTA, 0.5 mM MgCl_2_, 10% glycerol, protease inhibitor cocktail (Epizyme), phosphatase inhibitor cocktail (Epizyme), and 10 μM pervanadate (NEB), and then incubated with antibody-bound beads overnight at 4 °C. Finally, the antibody/protein complexes were washed with lysis buffer for five times, boiled with protein loading sample buffer, and subjected to western blot analysis.

#### Luciferase assay

Luciferase assay was performed as previously described using a Dual-Luciferase Reporter Assay System (Promega) according to the manufacturer's instructions [31, 32]. In detail, 8xGTIIC plasmid was used to detect the TEAD-associated transcriptional activity and Renilla plasmid was used as the internal transfection control. Whereas the pGL3 empty vector was used as a negative control. The transfection procedure was performed using Lipofectamine2000 (Invitrogen) following the manufacturer’s instructions.

#### Flow cytometry analysis

2 × 10^5^ BC cells were first trypsinized, harvested and washed with PBS buffer, and then single-cell suspensions were incubated in 400 μL running buffer (PBS + 5%FBS) with anti-human CD44 (BD company) and anti-human CD24 (BD company) on ice for 30 min. Finally, the percentages of labeled cells were analyzed using the CytoFLEX LX flow cytometer (Beckman).

### Immunohistochemistry (IHC) staining and in vitro proximity ligation assay

The IHC staining was performed as previously described [31] and the primary antibody for IHC was listed in the Supplementary table 1. In vitro proximity ligation assay was performed with the Duolink In Situ Orange Starter Kit Mouse/Rabbit (Sigma) according to the manufacturer’s instructions. Images were captured by Carl Zeiss confocal microscope (LSM 800) using a 40 × objective and analyzed with the image analysis software ZEN.

### CCK-8 cell proliferation, tumorsphere growth, cell migration and invasion assays

#### CCK-8 assay

Cell viability assay was performed using Cell Counting Kit-8 (Abbkine). Briefly, cells stably expressing the corresponding plasmids were seeded in triplicate in 96-well plates for indicated time, and then the OD value at 450 nm was detected according to the manufacturer’s instruction.

Tumorsphere growth, cell migration and invasion assays were conducted as previously described [31].

### In vivo xenograft assays.

All the animal-related protocols in this study were approved by the Institutional Animal Care and Use Committee of Sun Yat-Sen University. All the animal experiments were conducted following the approved protocols as we previously described, including limiting dilution assay (subcutaneous injection) and lung metastasis model (tail vein injection) [31].

### Human samples and tissue microarray

The human BC tissues used in this study for western blot were collected at the time of surgical resection in the Seventh Affiliated Hospital of Sun Yat-sen University. The human BC tissue microarrays used in this study were purchased from Shanghai Outdo Biotech, China. All the BC patients involved in this study provided informed consent, and this study was ethically approved by the Ethics Committee of the Seventh Affiliated Hospital of Sun Yat-sen University. The IHC scores were calculated based on the percentage of positively-stained cells and staining intensity, as previously described [31].

### RNA-sequencing and mass spectrometry analysis

The methods for RNA-sequencing and analysis were followed as we previously described [31]. For the Flag-IP-MS assay, the normal epithelial cells stably expressing 3xFlag-WT-YAP1 or 3xFlag-mut-YAP1 (3YF mutant) were generated by retrovirus infection. Cells were cultured to 80% confluency and lysed with a buffer containing protease/phosphatase inhibitor cocktails (Epizyme). Lysate was first incubated with anti-Flag antibody conjugated resin (Sigma) for 2 h at 4 °C and washed five times with lysis buffer. Bound proteins were then eluted with an elution buffer containing 200 ng/µl 3xFlag peptide. The eluate was further incubated with streptavidin conjugated resin for 2 h at 4 °C. Resin was washed and then eluted with a buffer containing 4 mM biotin. The purified YAP1 and associated proteins were trypsin digested for 16 h at 37 °C. Trifluoroacetic acid was added to the digestion solution to a final concentration of 0.5%, incubated at 37 °C for 45 min, and centrifuged at 13,000 rpm for 10 min. The top 90% of the sample after centrifugation was desalted and concentrated using a Millipore microC18 ZipTip and dried and resuspended in 0.1% formic acid. The resulting sample was analyzed by LC/ESI MS/MS using an Eksigent 2D nanoLC coupled in-line with an LTQ-OrbiTrap mass spectrometer. The details to identify proteins from mass spectrometry data were conducted as previously described [33].

### TCGA data analysis

*SRC* and *KLF5* mRNA expression data in human BC and adjacent tissues was downloaded from the online Ualcan Database (http://ualcan.path.uab.edu/analyisi.html). The expression patterns of YAP1 and KLF5 in BC subtypes and normal tissues were downloaded from the GEPIA2 Database (http://gepia.cancer-pku.cn/). Kaplan–Meier analysis of SRC and KLF5 in BC patients was conducted on the survival results and Kaplan–Meier survival curves were generated from the online Kaplan–Meier plotter Database (http://kmplot.com/analysis/index.php?p=service). Data are shown as means ± SD. P values were calculated with two-tailed unpaired Student’s t-test; *P < 0.05, **P < 0.01.

### Statistical analysis

All the in vitro experiments in this study were repeated at least three times in independent experiments. The graphs represent mean ± standard deviation (SD). The statistical analysis for comparison between two groups was performed by an unpaired Student's *t*-test. For multiple groups, one-way ANOVA followed by Tukey’s test was used as indicated in GraphPad Prism 5 software. *P* values < 0.05, or < 0.01, or < 0.001 were considered to be statistically significant and marked with *, ** and *** respectively. *P* > 0.05 was considered to be non-significant and marked as NS.

## Results

### SRC expression is prominently upregulated in TNBC tissues and cells, and highly associated with tumor malignancy

**Fig. 1:**
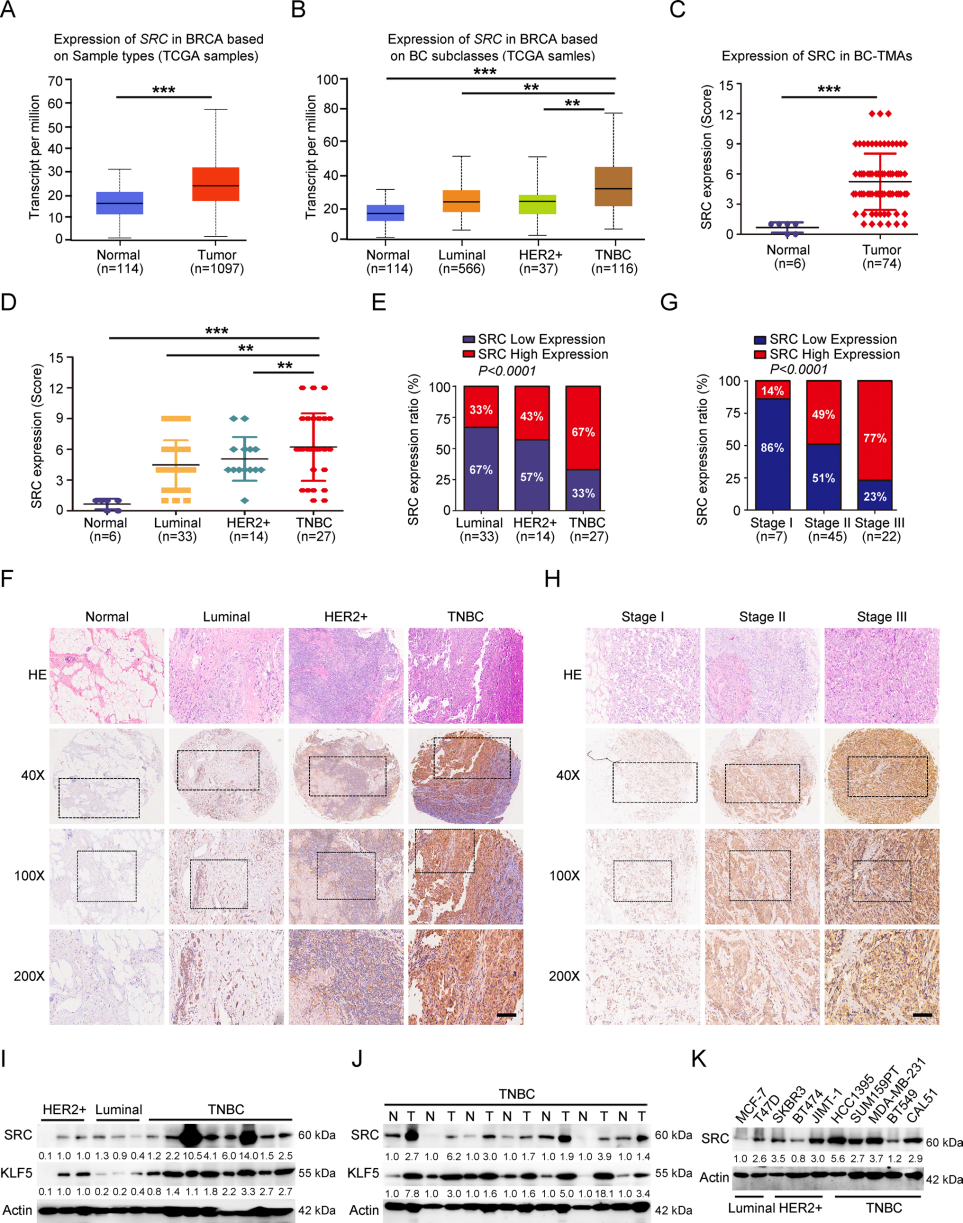
SRC expression in human BC tissues and cells. **A–B** Expressions of *SRC* mRNA in TCGA BC RNA-seq dataset including 114 normal and 1097 tumor tissues. **C–D** Expressions of SRC protein in 6 normal and 74 BC tissues based on SRC IHC scores. **E–F** Quantitation of SRC expression level in 74 BC tissues with different subtypes according to the IHC score. Representative images indicated the expression of SRC in different BC subtypes. Scale bars = 100 μm. **G–H** Quantitation of SRC expression level in 74 BC tissues with different TNM stages from I to III according to the IHC score. Representative images indicated the expression of SRC in different TNM stages of BCs. Scale bars = 100 μm. **I–K** Western blot analyses of total proteins from tumor tissues or BC cell lines with different subtypes using the indicated antibodies. The western-blot band intensities of SRC or KLF5 were normalized to the corresponding Actin intensity, and the quantitative values have been provided

To examine the associations of SRC expression level with the molecular subtypes of BCs, we first evaluated *SRC* mRNA expression in breast tumors from the cancer genome atlas (TCGA)-BRCA datasets, including 114 normal tissues and 1097 primary BC tumor tissues. The results revealed that *SRC* mRNA level was significantly upregulated in primary tumor tissues compared in normal tissues, and the TNBC patients showed overall higher SRC expression than the other BC subtypes, including Luminal and HER2 + subtypes (Fig. 1A–B). Further investigating the SRC protein expression by BC-TMAs (IHC score ≤ 6 was defined as low expression and a score > 6 was considered to be high expression according to the SRC IHC signals) showed that SRC expression was significantly higher in BC tissues compared in normal tissues (Fig. S1A–B and 1C). Based on the BC subtypes, we also observed that around 47% of the breast tissues had high levels of SRC expression, and its expression ratio with an IHC score > 6 in TNBC was significantly higher compared to the other subtypes (Fig. 1D–F). In addition, we noticed that SRC expression level was highly related to breast tumor node metastasis (TNM) stage, and the BC tissues at stage III usually had a higher IHC score (Fig. 1G–H). All these data demonstrated that SRC expression was upregulated in TNBC tissues and its expression level was positively correlated with the tumor malignancy. Subsequently, we also conducted western blot analysis with BC patient tissue lysates, including tumor lesions and adjacent tumor-free tissues. Consistent with the SRC expression in TMAs, SRC overall showed a higher expression level in TNBC patients compared with non-TNBC patients, as well as in the adjacent normal tissues (Fig. 1I–J and S10A–B). In addition, we detected SRC protein level in various types of BC cell lines, and found it was widely expressed in various BC cell lines. Even so, SRC also showed a relatively higher expression level in TNBC cell lines (Fig. 1K and S10C). Altogether, our results showed that SRC was highly expressed in the TNBC tissues and cells, and its expression level was correlated with BC malignancy.

### SRC-GOF enhances CSC-like properties, tumor growth and metastasis

**Fig. 2:**
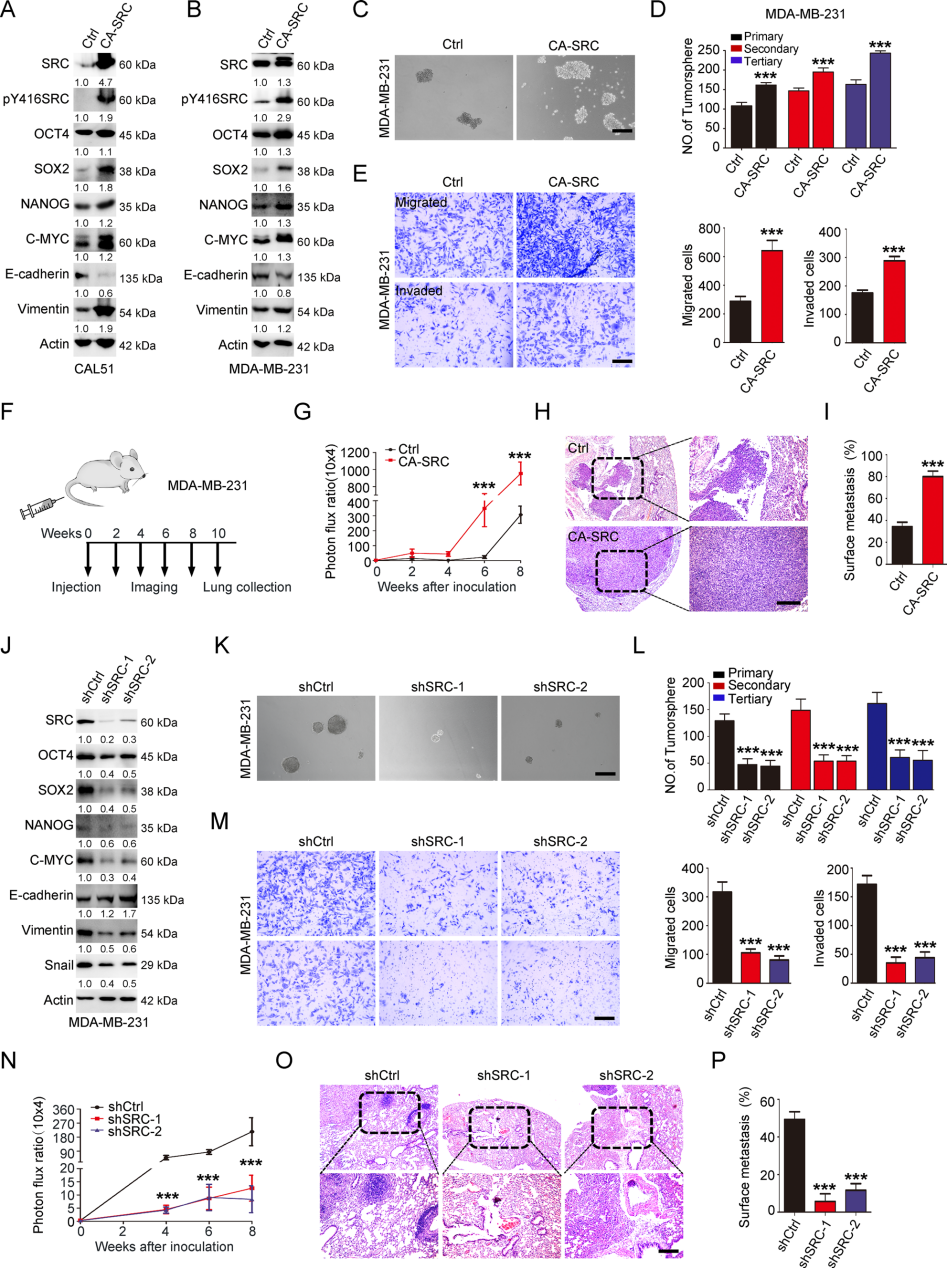
SRC regulates CSC-like properties, cell growth and migration/invasion behaviors. **A–B** Western blot analyses of total proteins from the CAL51 or MDA-MB-231 cells stably expressing vector control (Ctrl) or CA-SRC using the indicated antibodies. The western-blot band intensities of various markers were normalized to the corresponding Actin intensity, and the quantitative values have been provided. **C–D** Tumorsphere formation ability was analyzed in Ctrl or CA-SRC MDA-MB-231 cells. Representative images of tumorspheres were shown. Scale bars = 100 μm. The quantitation data represent means ± SD with 3 biological replicates. **E** In vitro cell migration/invasion ability was measured in Ctrl or CA-SRC MDA-MB-231 cells using the Transwell chamber or Transwell chamber containing the Matrigel as barrier. Representative images of migrated/invaded cells were shown. Scale bars = 100 μm. The quantitation data represent means ± SD with 3 biological replicates. **F** Schematic for tail vein injections of MDA-MB-231 cells, luciferase-labeled cells were captured and analyzed every two-weeks after the injection for 10 weeks. Mice were terminated and the lungs were collected for HE staining at the tenth week. **G** The quantification data was based on the bioluminescence signal intensities of lung-colonized tumor cells and represent means ± SD. **H–I** HE staining of sections from lung nodules and the quantification data represent the relative area of lung nodules. *n* = 6. Scale bar = 100 µm. **J** Western blot analyses of total proteins from the MDA-MB-231 cells stably expressing shRNA vector control (shCtrl) or shSRC using the indicated antibodies. The western-blot band intensities of various markers were normalized to the corresponding Actin intensity, and the quantitative values have been provided. **K–L** Tumorsphere formation ability was analyzed in shCtrl or shSRC MDA-MB-231 cells. Representative images of tumorspheres were shown. Scale bars = 100 μm. The quantitation data represent means ± SD with 3 biological replicates. **M** In vitro cell migration/invasion ability was measured in shCtrl or shSRC MDA-MB-231 cells using the Transwell chamber or Transwell chamber containing the Matrigel as barrier. Representative images of migrated/invaded cells were shown. Scale bars = 100 μm. The quantitation data represent means ± SD with 3 biological replicates. **N** The quantification data was based on the bioluminescence signal intensities of lung-colonized tumor cells and represent means ± SD. **O–P** HE staining of sections from lung nodules and the quantification data represent the relative area of lung nodules. *n* = 6. Scale bar = 100 µm

To explore the detailed function of SRC kinase in TNBC, we conducted the GOF assays using lentivirus to stably express constitutively activated SRC (CA-SRC: Y530F) in both MCF10A and TNBC cells. Western blot analysis in these cells revealed that CA-SRC dramatically induced the expression of SOX2. Meanwhile, other cancer stemness-related markers, such as OCT4, NANOG and C-MYC were also slightly induced after SRC activation (Fig. 2A–B, S2L–M, S10D–E and S13C–D). In addition, epithelial-mesenchymal transition (EMT)-associated marker E-cadherin was repressed, while Vimentin was enhanced in SRC-GOF cells (Fig. 2A–B, S2M, S10D–E and S13D), revealing the potential roles for SRC kinase in cancer stemness and EMT regulations. We and others have previously proved that upregulation of stemness and EMT-related markers could confer tumor cells with CSC-like properties, and CD44^+^/CD24^−/low^ cells were widely used to characterize the BCSC populations or tumor-initiating cells [34]. Thus, to examine the potential effect of SRC activation on CSC-like properties, we conducted the flow cytometry analysis using CD44/CD24 antibodies. The result revealed that CA-SRC significantly increased the percentage of CD44^+^/CD24^−/low^ population in CAL51 cells (Fig S2A-B). In addition, tumorsphere formation assay was performed to assess the BCSC self-renewal in vitro. We observed that CA-SRC dramatically induced the tumorsphere growth ability derived from the TNBC cells, as well as the mammosphere formation efficiency of MCF10A cells (Fig. 2C–D, S2C and 2 N). All these data confirmed that SRC activation could indeed enhance the cancer stemness in TNBC cells. Owing that enhanced cancer stemness is closely correlated to the tumor cell growth and metastasis abilities, we then performed the CCK-8 and Transwell assays to analyze the cell proliferation, migration and invasion abilities upon SRC kinase activation. As anticipated, CA-SRC in TNBC cells dramatically induced the cell proliferation rate, as well as the migratory and invasive capabilities compared with the control cells (Fig. 2E and S2D–F). Consistently, the in vivo xenograft assay also showed that CAL51 cells with CA-SRC remarkably induced the tumor cell growth and size, as evidenced by increased tumor weight and Ki67 IHC signals in CA-SRC tumors (Fig. S2G–J). Utilizing a xenograft metastasis model via tail vein injection of MDA-MB-231 cells (Fig. 2F), we discovered that CA-SRC cells could form more pulmonary localizations, and larger lung metastasis nodules than the control cells in vivo (Fig. 2G–I and S2K). Taken together, our results demonstrated that SRC-GOF enhanced cancer stemness, tumor cell growth and metastasis in TNBCs.

### SRC-LOF reduces CSC-like properties, tumor growth and metastasis

To validate the phenotypes we observed above in SRC-GOF TNBC cells, we further performed the SRC-LOF studies using short hairpin RNAs (shRNAs)-mediated SRC knockdown technology. As anticipated, SRC downregulation obviously inhibited the expressions of CSC-associated markers and the EMT program (Fig. 2J, S3A, S10F and S14A). Flow cytometry analysis of the BCSC population and tumorsphere growth assay also revealed that downregulation of SRC dramatically reduced the percentage of CD44^+^/CD24^−/low^ population, and the tumor cell self-renewal ability (Fig. 2K–L and S3B-C). Moreover, we also performed the limiting dilution assay using the SRC stable knockdown CAL51 cells, to evaluate the tumor-initiating frequency in vivo. The xenograft assay also showed that the decrease of SRC was indeed able to reduce tumor incidence (Fig S3D–F). Subsequently, we also did the CCK-8 and Transwell assays to evaluate the tumor cell proliferation, migration/invasion abilities upon SRC downregulation. The results showed that SRC-LOF in TNBC cells significantly restrained these cell behaviors in vitro (Fig. 2M and S3G–I). More importantly, in vivo xenograft experiments also displayed that SRC knockdown TNBC cells significantly attenuated the tumor growth and size, as well as the pulmonary localization ability compared to the control cells (Fig. 2N–P and S3J-N). Dasatinib, a representative SFK inhibitor, has shown anti-tumor effects on human cancer cells both in vitro and in vivo. Here, we also found that Dasatinib treatment dramatically inhibited BCSC properties in TNBC cells, including CD44^+^/CD24^−/low^ population and tumorsphere growth, as well as cell proliferation in vitro (Fig S4A–F). Taken together, all our results suggested that SRC regulated the cancer stemness, tumor cell growth and metastasis in TNBC cells.

### SRC regulates YAP1 activation independently of the canonical hippo kinases

**Fig. 3:**
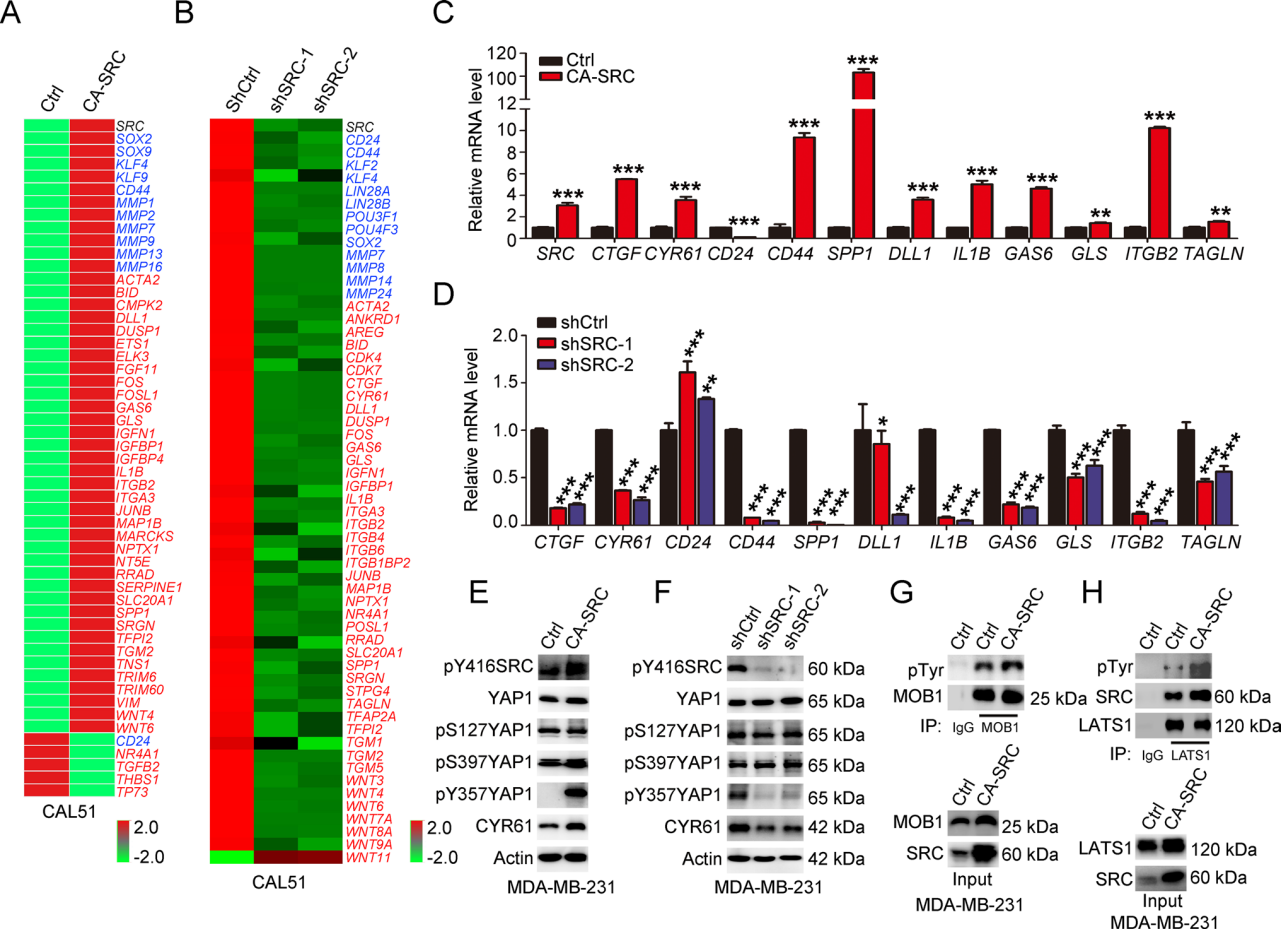
SRC directly activates YAP1 independently of canonical Hippo kinases. **A–B** The heatmap indicated the gene expression changes induced by overexpression of CA-SRC or SRC knockdown in CAL51 cells. Blue color indicated the cancer stemness and metastasis-related genes, and the red color indicated the known YAP1 target genes in BC cells. **C–D** Quantitative real-time PCR to examine the mRNA level of the indicated genes in CA-SRC or SRC knockdown CAL51 cells. The data are shown as the mean ± SD (*n* = 3). Statistically significant differences were indicated. **E–F** Western blot analyses of total proteins from the MDA-MB-231 cells stably expressing Ctrl, CA-SRC or shSRC using the indicated antibodies. **G–H** Western blot analyses of LATS1 and MOB1 tyrosine phosphorylation level in MDA-MB-231 cells stably expressing Ctrl and CA-SRC using p-Tyr antibody after LATS1 or MOB1 immunoprecipitation

To clarify the molecular mechanism of SRC kinase-regulated phenotypes in TNBC cells, RNA-sequencing was conducted using both SRC-GOF and -LOF CAL51 cells. The analysis of differentially expressed genes (DEGs) in SRC activated cells showed that total 4378 genes were down- or upregulated with log2-fold change > 2 and *p* < 0.05, while 6990 common DEGs were identified in SRC knockdown cells with two different SRC shRNAs (Fig. S5A–C). Further characterization of these common DEGs revealed that stemness and metastasis-associated markers, including *CD44*, *SOX2*, *KLF4* and some MMP family member genes, were regulated by SRC kinase (Fig. 3A–B). More interestingly, we found that a substantial number of Hippo-YAP1 downstream target genes reported in BC cells [35–38], including *CYR61*, *CTGF*, *ETS1*, *FOSL1*, *JUNB*, were significantly increased upon SRC activation, while dramatically decreased in SRC knockdown cells (Fig. 3A–B). Gene set enrichment analysis also showed that YAP/TAZ signature was enriched in SRC-regulated transcriptome and YAP/TAZ signature indeed ranked as the top in SRC-regulated oncogenic program (Fig S5D-E). Further validation by qRT-PCR analysis indicated that YAP1-associated transcription was regulated by SRC kinase in TNBC cells (Fig. 3C–D). In addition, luciferase assay using the 8xGTIIC reporter also demonstrated that SRC activation could promote the TEAD-associated transcriptional activity, resembling with the effect by overexpression of YAP1 in these cells (Fig S5F). Co-transfection of CA-SRC and YAP1 showed a synergistic effect on the luciferase activity, as evidenced by comparison with either CA-SRC or YAP1 single overexpression group (Fig. S5F). Taken together, all these data indicated that SRC directly regulated YAP1/TEAD-associated transcriptional outputs in TNBC cells. As the key downstream effector of Hippo signaling, our previous study has revealed that YAP1 regulated cancer stemness and tumor progression in TNBC cells [31]. Meanwhile, another study also revealed that SRC activation was able to inhibit the Hippo pathway by tyrosine phosphorylation of LATS1, thereby leading to YAP1 activation [20]. Here, to elucidate whether SRC-mediated YAP1 activation was dependent on canonical Hippo kinases in TNBC cells, we firstly examined YAP1 phosphorylation status at S127 and S397 (YAP1 isoform with 504aa) by western blot. The results showed that the phosphorylation levels of these two sites were not affected in TNBC cells expressing either CA-SRC or SRC shRNAs, while the YAP1 downstream target CYR61, was fluctuating resembling SRC kinase, suggesting that SRC-mediated activation of YAP1 did not involve in the changes of canonical Hippo kinase cascade. On the contrary, the phosphorylation level of YAP1 at Y357, was dramatically induced in SRC-activated cells, while repressed in SRC knockdown TNBC cells (Fig. 3E–F, S5G, S11A and 14B). To further validate this conclusion, we directly analyzed the phosphorylation level of LATS1 and MOB1 in CA-SRC TNBC cells by western blot. We indeed observed that LATS1 tyrosine phosphorylation level was enhanced in SRC activated cells, while MOB1 tyrosine phosphorylation and LATS1 serine phosphorylation levels were not affected with SRC activation (Fig. 3G–H, S5H–J, S11B and S14C). Meanwhile, we also noticed that SRC activation dramatically induced the phospho-AKT activity, which was also reported to be a negative regulator of YAP1 (Fig S5J and 14D). Moreover, we found SRC-induced TEAD-associated transcriptional activity was not dependent on LATS kinase, owing to the increased luciferase activity by CA-SRC in LATS1/2 knockdown cells (Fig S5K). Taken together, all these data demonstrated that SRC directly activated YAP1 through tyrosine phosphorylation in TNBC cells, rather than in a Hippo kinase-dependent manner.

### YAP1 tyrosine phosphorylation enhances its interaction with transcription factor KLF5

**Fig. 4:**
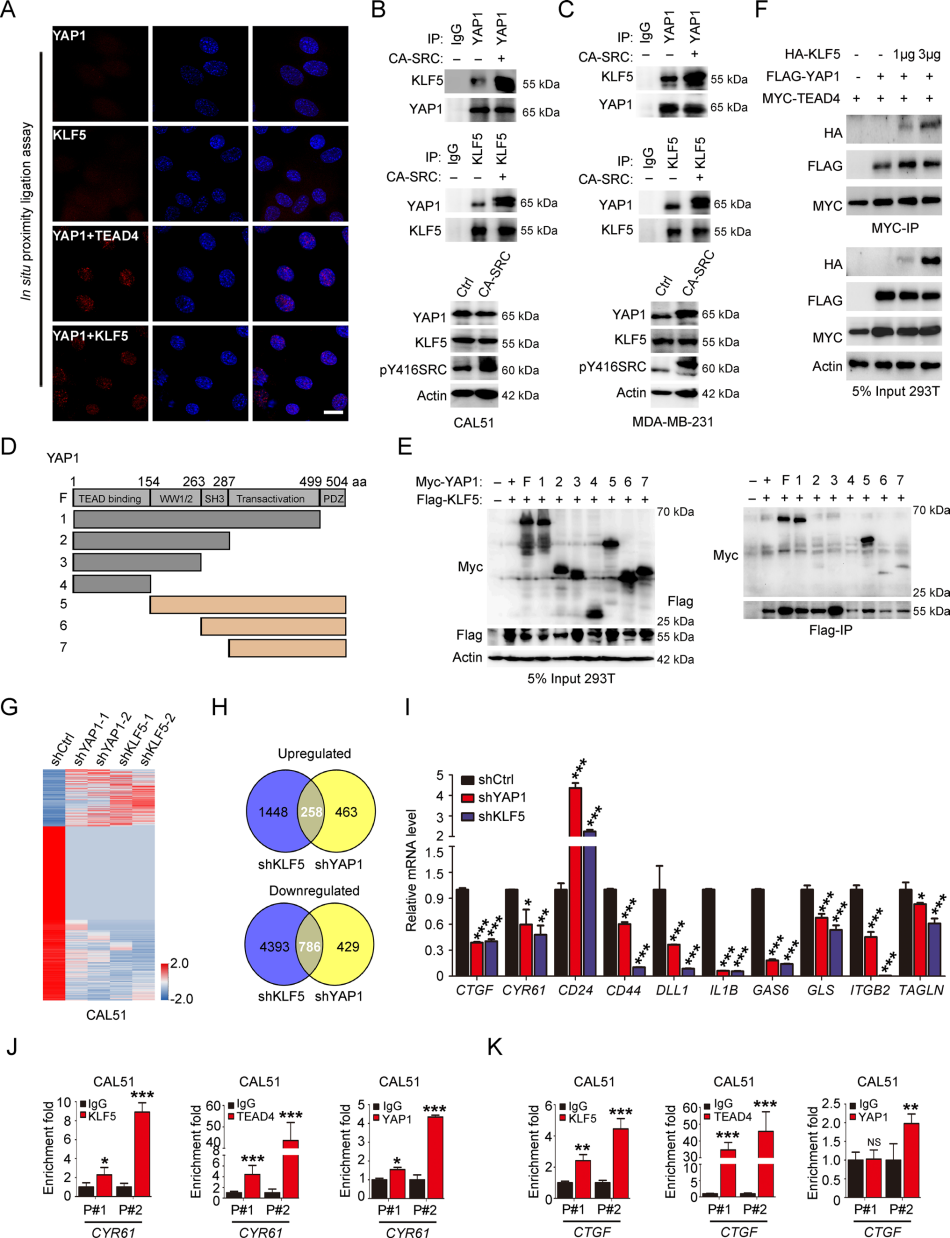
Identification of a SRC kinase-dependent YAP1-KLF5 interacting module. **A** In situ proximity ligation assay showing an interaction between YAP1 and KLF5 in the nucleus of MCF10A cells indicated by red fluorescent spots (the blue color indicated the DAPI stain). YAP1 or KLF5 antibody alone was used as negative control, and YAP1-TEAD4 interaction signals were used as the positive control. Scale bar = 100 µm. **B–C** Co-IP showing the interaction between exogenous YAP1 and KLF5 in CAL51 and MDA-MB-231 cells stably expressing Ctrl or CA-SRC plasmids. **D–E** Identification of the molecular motifs on YPA1 required for its interactions with KLF5 through Flag/Myc-IP and western blot analysis with the indicated antibodies in 293 T cells. **F** 293 T cells were transfected with fixed amounts of Myc-TEAD4 and Flag-YAP1, and increasing amounts of HA-KLF5, followed by Co-IP and western blot analyses with the indicated antibodies. **G–H** The heatmap indicated the gene expression changes induced by knockdown of KLF5 or YAP1 in CAL51 cells. The commonly upregulated or downregulated gene numbers identified by RNA-seq were indicated. **I** Quantitative real-time PCR to examine the mRNA level of the indicated genes in YAP1 or KLF5 knockdown CAL51 cells. The data are shown as the mean ± SD (*n* = 3). Statistically significant differences were indicated. **J-K** ChIP-qPCR assay showed that KLF5 and YAP1-TEAD4 co-occupied on the regulatory regions of *CYR61* and *CTGF* in CAL51 cells

YAP1 phosphorylation at tyrosine has been revealed to be an essential context for its interaction with its DNA-binding partners, including TBX3/5 and P73 [39–41]. Here, to identify novel YAP1 tyrosine phosphorylation-dependent interacting transcriptional factors, we first generated YAP1 knockout epithelial cells, and then rescued these cells with either Flag-labeled WT-YAP1 (isoform with 504aa), or 3Y to F/E mutant YAP1 (Y391/407/444F or E corresponds to Y341/357/394F or E in YAP1 isoform with 454aa) to analyze the cell transformation abilities (Fig S6A and 15A). The knockdown of endogenous YAP1 dramatically decreased MCF10A cell proliferation without EGF growth factor, while overexpression of sgRNA-resistant human WT-YAP1 could enhance the cell growth in 2D and 3D condition. However, overexpression of the 3YF mutant-YAP1 failed to show the above phenotypes, and the 3YE mutant-YAP1 showed a very similar efficiency as the WT-YAP1 in 2D growth, and a stronger transformation ability than WT-YAP1 in 3D condition (Fig. S6B–C). Subsequently, we performed a proteomic screen by mass spectrometry using Flag-labeled WT or 3YF mutant-YAP1 immunoprecipitated from SRC-activated cells (Fig. S6D). We screened the Flag-immunoprecipitation for candidates and found that Kruppel family member KLF5 was one of the most potential candidate transcription factors specifically interacting with WT -YAP1 based on the binding score with 1% FDR and q-value validation (< 0.05) (Fig. S6E–F). In our data, we also observed that YAP1-P73 interacting efficiency was decreased in mutant-YAP1 immunoprecipitates compared with in WT-YAP1 immunoprecipitates, while the YAP1 and C-JUN interaction was similar between WT and mutant -YAP1 immunoprecipitates (Fig S6F). Further validation of the mass spectrometry data by Flag-IP and western blot assays revealed that mutations of YAP1 tyrosine residues targeted by SRC kinase prominently reduced YAP1-KLF5 interaction efficiency (Fig. S6G and 15B). To further confirm this association, we conducted the in situ proximity ligation assays in CA-SRC cells and found that YAP1 in close proximity with KLF5 in the cell nuclear, resembling with the YAP1-TEAD4 interacting module in these cells (Fig. 4A). Subsequently, the Co-IP assays also showed that SRC kinase activation could robustly enhance both the YAP1-KLF5 interactions in 293 T cells, and also their endogenous interactions in TNBC cells (Fig. 4B–C, S6H, 12A and 15C). Further mapping of the YAP1-KLF5 interacting motifs revealed that both the SH3 domain and transactivation domain of YAP1 were indispensable for their interactions (Fig. 4D–E and S12B). More interestingly, we discovered that YAP1-KLF5 module could co-bind to TEAD protein, and their binding to TEAD was not mutually exclusive (Fig. 4F and S12C). Then to investigate the biological outcomes of SRC-mediated KLF5-YAP1/TEAD associations, we firstly tested the effect of KLF5 overexpression on TEAD-related luciferase activity. The results showed that KLF5 was able to promote the luciferase activity, resembling YAP1 overexpressing in these cells (Fig. S6I), whereas knockdown of KLF5 led to the reduced TEAD-dependent transcriptional activity (Fig S6J). Moreover, co-overexpression of WT-YAP1, rather than Y-F mutant YAP1, together with KLF5 synergistically boosted TEAD-mediated transcriptional activity (Fig. S6K). In addition, the DEG analyzing in KLF5 and YAP1 knockdown cells obtained from RNA-sequencing showed that 6885 genes were down- or upregulated with more than two-fold changes in KLF5 knockdown cells, while only 1936 DEGs were identified in YAP1 knockdown cells (Fig. S6L–M). Further characterization of these common DEGs in both KLF5 and YAP1 knockdown cells revealed that 258 and 786 genes respectively were up or downregulated (Fig. 4G–H). Further validation by qRT-PCR revealed that SRC-induced genes were significantly down-regulated in YAP1 and KLF5 knockdown TNBC cells (Fig. 4I and S6N). Furthermore, ChIP-qPCR assay also showed that KLF5-YAP1/TEAD co-bond to the regulatory regions of YAP1 downstream genes, including *CYR61* and *CTGF* (Fig. 4J–K). However, SRC-induced YAP1-KLF5 module was not required for the transcription of the *KLF5* gene (Fig. S6O–P). Taken together, all these findings demonstrated that KLF5 could interact with YAP1-TEAD to enhance the TEAD-dependent transcriptional outputs.

KLF5 has demonstrated to be highly expressed in basal-type poorly differentiated BCs and its high expression is an unfavorable prognostic marker of BC patients [42, 43]. Moreover, previous studies showed that YAP1 promoted BC cell growth by stabilizing the KLF5 protein [44, 45]. We previously have found that YAP1 regulated the cancer stemness and metastasis in TNBC cells [31]. Here we further generated the YAP1 and KLF5-LOF TNBC cells and performed the in vivo xenograft experiments via tail vein injection of these cells. The results showed that KLF5 knockdown dramatically reduced the TNBC cell localizations and growth in lung, resembling the phenotypes observed in YAP1-LOF TNBC cells (Fig. S7A–H). These data suggested that YAP1 and KLF5 indeed performed very similar functions in TNBC cells.

### YAP1-KLF5 oncogenic module is responsible for SRC-induced CSC-like properties, tumor growth and metastasis

**Fig. 5:**
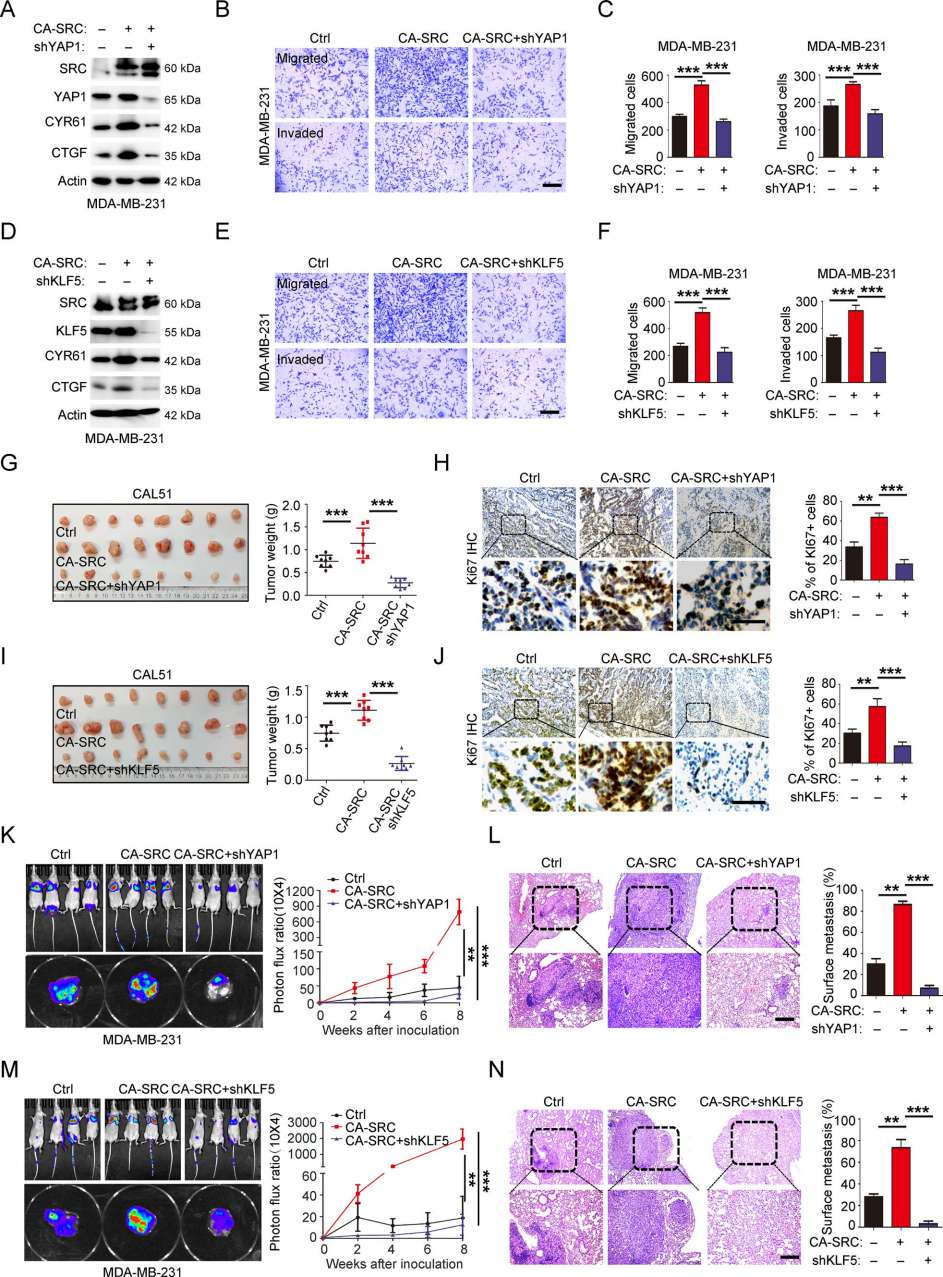
YAP1-KLF5 oncogenic module is responsible for SRC-enhanced CSC-like properties, cell growth and migration/invasion behaviors. **A** Western blot analyses of total proteins from MDA-MB-231 cells stably expressing Ctrl, CA-SRC or CA-SRC + shYAP1 using the indicated antibodies. **B–C** In vitro cell migration/invasion ability was measured in MDA-MB-231 cells stably expressing Ctrl, CA-SRC or CA-SRC + shYAP1 using the Transwell chamber or Transwell chamber containing the Matrigel as barrier. Representative images of migrated cells were shown. Scale bars = 100 μm. The quantitation data represent means ± SD with 3 biological replicates. **D** Western blot analyses of total proteins from MDA-MB-231 cells stably expressing Ctrl, CA-SRC or CA-SRC + shKLF5 using the indicated antibodies. **E–F** In vitro cell migration/invasion ability was measured in MDA-MB-231 cells stably expressing Ctrl, CA-SRC or CA-SRC + shKLF5 using the Transwell chamber or Transwell chamber containing the Matrigel as barrier. Representative images of migrated cells were shown. Scale bars = 100 μm. The quantitation data represent means ± SD with 3 biological replicates. **G–J** Xenograft tumor formation assays in NOD-SCID mice using CAL51 cells stably expressing Ctrl, CA-SRC or CA-SRC + shYAP1 or shKLF5. Quantitation of tumor weight represent means ± SD with *n* = 8. Representative images for Ki67 IHC and quantitation data were shown with means ± SD. Scale bars = 100 μm. **K–N** Bioluminescence images of lung-colonized tumor cells injected through the tail vein using NOD/SCID mice at the tenth week (*n* = 6 per group), the quantification data was based on the bioluminescence signal intensities and represent means ± SD. HE staining of sections from lung nodules and the quantification data represent the relative area of lung nodules. *n* = 6. Scale bar = 100 µm

To clarify whether activation of the YAP1-KLF5 oncogenic module is responsible for the phenotypes we observed in SRC-GOF cells, we generated the CA-SRC TNBC cells, and followed by either stable knockdown of YAP1 or KLF5 using the shRNAs. Firstly, we confirmed that decrease of YAP1 or KLF5 expression in SRC-activated TNBC cells could attenuate the expressions of YAP1 downstream targets, including CYR61 and CTGF (Fig. 5A and D, and S13A-B). Further analyzing the percentages of CD44^+^/CD24^−/low^ population and the tumorsphere growth ability revealed that knockdown of either YAP1 or KLF5 in CA-SRC TNBC cells was able to rescue SRC-enhanced CSC-like properties (Fig. S8A–D). We further investigated the effect of YAP1-KLF5 module disruption on SRC-induced cell growth, cell migration/invasion behaviors in vitro and in vivo. CCK-8 and xenograft assay results showed that disruption of this module in SRC-activated TNBC cells could potentially inhibit the hyperproliferation in vitro (Fig. S8E–F) and the tumor overgrowth in vivo (Fig. 5G–J). Besides, the Transwell assay and xenograft metastasis assay also demonstrated that attenuation of YAP1-KLF5 association in SRC-activated TNBC cells dramatically repressed their migration and invasion capacities in vitro (Fig. 5B–F and S8G–H), as well as their pulmonary localization abilities in vivo (Fig. 5K–N). Taken together, all the above findings revealed that YAP1-KLF5 module functioned as the key downstream regulator of SRC kinase in TNBC cells to regulate BC stemness, growth and metastasis.

### SRC-YAP1/KLF5 signaling axis prognosticates the malignance of TNBC patients

**Fig. 6:**
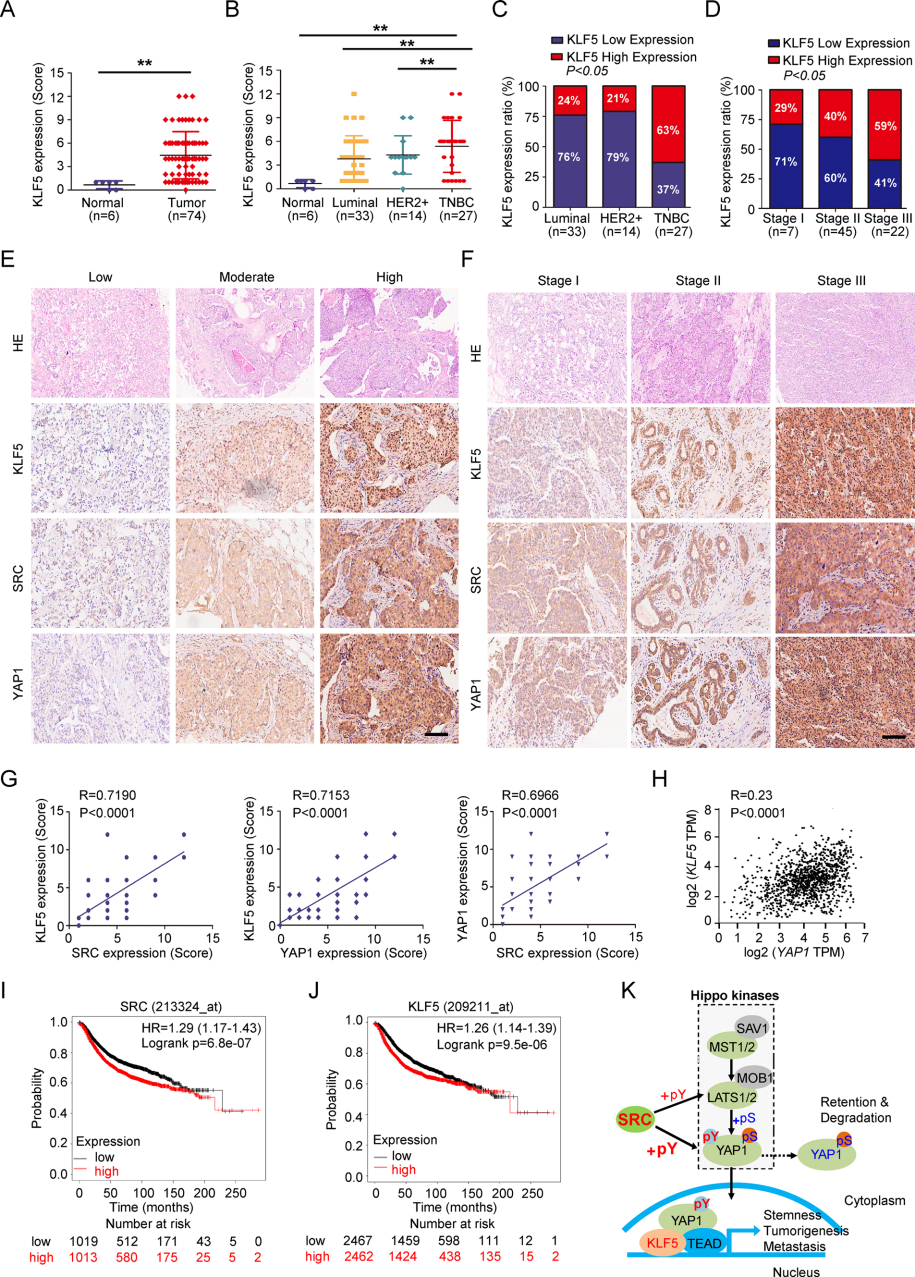
Clinical relevance of SRC-YAP1/KLF5 signaling axis with human BCs. **A–B** KLF5 protein expression levels in 6 normal and 74 BC tissues were detected by IHC and analyzed by IHC scores. **C–D** Quantitation of KLF5 expression level in 74 BC tissues with different subtypes or TNM stages according to the IHC scores. **E–F** Representative images indicated the expression level of SRC, YAP1 or KLF5 in BC tissues or with different TNM stage from I to III according to the IHC scores. Scale bars = 100 μm. **G–H** Positive correlation of SRC with YAP1-KLF5 module was assessed using Pearson correlation coefficient analysis according to the corresponding IHC scores. **I–J** Kaplan–Meier analysis of overall survival curves for BC patients grouped according to SRC or KLF5 expression level. **K** Schematic of the proposed model to illustrate that SRC-YAP1/KLF5 regulatory axis induces the CSC properties, tumor growth and metastasis in TNBC independently of canonical Hippo kinases

To assess the potential relevance of SRC-YAP1/KLF5 signaling axis with the BC patients, we further performed the TMA analysis using the YAP1 and KLF5 antibodies as we previously have done for analyzing SRC expression in patient BC tissues (Fig S1A). As we observed, like SRC IHC staining, YAP1 and KLF5 also showed significantly higher expression levels in BC tissues based on the YAP1/KLF5 IHC scores (Fig. 6A and S9A). Meanwhile, both of them showed prominently higher expression level in TNBC tissues compared in other BC subtypes, including luminal and HER2 + BCs (Fig. 6B–C and S9B-C). In addition, both YAP1 and KLF5 protein expression levels were significantly related to the BC TNM stage, owing that both YAP1 and KLF5 have relatively higher IHC scores and ratios in BC tissues at Stage III (Fig. 6D and S9D). Furthermore, the expression of SRC in these tissues was positively associated with YAP1-KLF5 module, and co-expressions of SRC and YAP1-KLF5 module were significantly correlated to the TNM stage and tumor malignancy in BCs (Fig. 6E–H). Consistent with our findings, although the mRNA expression level of *KLF5* in BCs was lower than that in normal tissues, its protein expression was significantly higher in BCs based on the analysis of the TCGA database (Fig. S9E–F). Moreover, we found that BC patients with SRC or KLF5 high expression also suffered shorter overall survival based on the TCGA-BRCA datasets (Fig. 6I–J). Taken together, all our findings indicated the clinical relevance of the SRC-YAP1/KLF5 signaling axis in BC patients. Therefore, targeting the YAP1-KLF5 module may represent a rational therapeutic strategy for SRC aberrantly activated TNBCs.

## Discussion

In our present study, we demonstrate the tyrosine kinase SRC directly regulated a YAP1-KLF5 oncogenic module through YAP1 tyrosine residue phosphorylation, and independently of the canonical Hippo kinases (Fig. 6K). These results, therefore, revealed a novel SRC-YAP1/KLF5 regulatory axis to induce the cancer stemness and metastasis in TNBC cells.

The SRC kinase is a well-known therapeutic target for various types of cancers, including BCs. However, the impact of SRC inhibition appears different among subtypes of BC cells in multiple preclinical studies. For instance, inhibition of SRC kinase activity with Dasatinib decreases the growth of TNBC cells without clear effect on other BC subtypes [27]. Furthermore, Dasatinib sensitizes TNBC cells to chemotherapy by targeting BCSCs [28], and its antitumor effect is highly associated with a decreased proportion of aldehyde dehydrogenase 1-positive cells [29]. These studies indicated the essential role of SRC kinase in BCSCs. However, the role of SRC kinase in BCSC stemness regulation and the underlying mechanism are rarely reported. TNBCs are enriched in BCSCs that are characterized by expression of cell surface marker CD44^high^CD24^low^, and which may contribute to the recurrence and metastasis of TNBC patients [6]. Here we have demonstrated that SRC kinase was prominently expressed in TNBC patients, and it was a BCSC stemness regulator in TNBC cells, which may address the issue that TNBCs are more sensitive to the SRC inhibitor than the other BC subgroups. We further identified the YAP1-KLF5 oncogenic module was responsible for SRC-induced cancer stemness in TNBC cells, targeting YAP1-KLF5 module thus may represent a more precise therapeutic strategy for treating SRC activation-associated TNBC.

Hippo signaling plays essential roles in cell growth and organ size control, and its dysregulation thus involves in the tumorigenesis [46, 47]. The canonical Hippo signaling consists of MST1/2 kinases that directly phosphorylate LATS1/2. LATS1/2 then phosphorylates YAP1 to sequester it into the cytoplasm, where it could be firstly marked by 14–3-3 protein, and then degraded by ubiquitination-mediated pathway [33]. Inhibition of this pathway will allow YAP1 shuttle to the nucleus where it forms a transcriptional complex with TEAD family members to regulate the downstream genes [48, 49]. SRC plays a central role by modulating several important signaling pathways, such as PI3K-AKT, RAS-MAPK and Hippo signaling [50]. Here, we identified YAP1 as the key downstream substrate of SRC-induced cancer stemness and metastasis in TNBC cells. YAP1 was firstly identified as Yes-associated protein [51], and later it was shown to be tyrosine-phosphorylated by SFKs, including YES, ABL and SRC [52, 53]. Although both our current work and previous study have shown that SRC kinase could inhibit the Hippo tumor suppressor pathway through tyrosine phosphorylation of LATS1 in a YAP1-dependent manner, we still observed the increased TEAD-associated transcriptional activity in LATS1/2 knockdown cells. Our study thus suggested that SRC-mediated YAP1 regulation was not associated with the canonical Hippo kinases. In line with our findings, SRC-mediated YAP1 activation through direct phosphorylation has been implicated in many cancer progressions and tumor microenvironment regulation [32]. For example, phosphorylation of YAP1 by the YES is necessary for survival and tumorigenesis β-catenin-driven cancers, which are sensitive to the SRC inhibitor Dasatinib [40]. Similarly, tyrosine phosphorylation of YAP1 by SRC is also required for RASSF1C overexpression-induced cell motility, invasion, and tumorigenesis [41]. In addition, SRC-dependent YAP1 activation has been demonstrated to be critical for the establishment and maintenance of cancer-associated fibroblasts [54]. Therefore, YAP1 tyrosine phosphorylation represents a potential mechanism for SRC activation-associated human cancers.

Due to lack of a DNA-binding domain, TEAD family transcriptional factors are required to mediate YAP1-associated transcriptional output [47, 55]. However, they are not the sole members, and not sufficient to execute all transcriptional programs in different contexts. Accumulating studies have demonstrated that cellular context has a pivotal role in the choice of the YAP1/TAZ partners and consequently on the final transcriptional outputs [56]. YAP1 tyrosine phosphorylation mediated by SRC family kinase has demonstrated to be a critical context for the recruitment of YAP1-interacting proteins, like RUNX2, P73 and TBX3/5 [39–41, 53]. In our study, we have also identified KLF5 as a YAP1 tyrosine phosphorylation-dependent interacting transcriptional factor to mediate SRC activation-enhanced cancer stemness and metastasis in TNBCs. Therefore, targeting YAP1-KLF5-mediated transcriptional output may represent a more precise strategy for TNBC treatment induced by SRC aberrant activation.

### Supplementary Information

Below is the link to the electronic supplementary material.

Supplementary file1 (DOCX 20 KB)

Supplementary file2 (DOCX 24 KB)

Supplementary file3 (XLSX 17 KB)

Figure S1. SRC expression in human BC tissues and cells. (A). The whole slide scans for SRC IHC stain with 6 normal tissues and 74 BC tissues. 6 normal tissues have been indicated as “N” in the figure and all the others are BC tissues indicated with “T”. (B). Representative images indicated the low (score ≤6) and high (score >6) expression level of SRC in BC tissues detected with IHC. Scale bars=100 μm. Supplementary file4 (TIF 16393 KB)

Figure S2. SRC-GOF enhances CSC-like properties, cell growth and migration/invasion behaviors. (A-B). Representative images showed the populations of BCSCs (CD44+/CD24-/low) analyzed by flow cytometry in CAL51 cells stably expressing Ctrl or CA-SRC. The quantitation data represent means ± SD with 3 biological replicates. (C). Tumorsphere formation ability was analyzed in Ctrl or CA-SRC CAL51 cells. Representative images of tumorspheres were shown. Scale bars=100 μm. The quantitation data represent means ± SD with 3 biological replicates. (D-E). Cell viability was measured by CCK-8 assay in CAL51 or MDA-MB-231 cells stably expressing Ctrl or CA-SRC. The quantitation data represent means ± SD with 3 biological replicates. (F). In vitro cell migration/invasion ability was measured in Ctrl or CA-SRC CAL51 cells using the Transwell chamber or Transwell chamber containing the Matrigel as barrier. Representative images of migrated/invaded cells were shown. Scale bars=100 μm. The quantitation data represent means ± SD with 3 biological replicates. (G-J). Xenograft tumor formation assays in NOD-SCID mice using CAL51 cells stably expressing Ctrl or CA-SRC. IHC analyses of Ki67 protein expression in CAL51 cell-derived xenograft tissues. Scale bars=100 μm. Quantitation of tumor weight or Ki67+ cells represent means ± SD. (K). Bioluminescence images of lung-colonized tumor cells injected through the tail vein using NOD/SCID mice at the tenth week (n=6 per group). (L-M). Western blot analyses of total proteins from the MCF10A cells stably expressing Ctrl or CA-SRC using the indicated antibodies. (N). Mammosphere formation efficiency was determined in Ctrl or CA-SRC MCF10A cells. Representative images of mammospheres were shown. Scale bars=100 μm. The quantitation data represent means ± SD with 3 biological replicates. Supplementary file5 (TIF 14957 KB)

Figure S3. SRC-LOF reduces CSC-like properties, cell growth and migration/invasion behaviors. (A). Western blot analyses of total proteins from the CAL51 cells stably expressing Ctrl or CA-SRC using the indicated antibodies. The western-blot band intensities of various markers were normalized to the corresponding Actin intensity, and the quantitative values have been provided. (B). Representative images showed the populations of BCSCs (CD44+/CD24-/low) analyzed by flow cytometry in CAL51 cells stably expressing shCtrl or shSRC. The quantitation data represent means ± SD with 3 biological replicates. (C). Tumorsphere formation ability was analyzed in shCtrl or shSRC TNBC cells. Representative images of tumorspheres were shown. Scale bars=100 μm. The quantitation data represent means ± SD with 3 biological replicates. (D-F). Tumor-initiating cell frequency was analyzed by in vivo limiting dilution assay using CAL51 cells stably expressing shCtrl or shSRC, n=8. (G-H). Cell viability was measured by CCK-8 assay in CAL51 or MDA-MB-231 cells stably expressing shCtrl or shSRC. The quantitation data represent means ± SD with 3 biological replicates. (I). In vitro cell migration/invasion ability was measured in shCtrl or shSRC CAL51 cells using the Transwell chamber or Transwell chamber containing the Matrigel as barrier. Representative images of migrated/invaded cells were shown. Scale bars=100 μm. The quantitation data represent means ± SD with 3 biological replicates. (J-M). Xenograft tumor formation assays in NOD-SCID mice using CAL51 cells stably expressing shCtrl or shSRC. IHC analyses of Ki67 protein expression in CAL51 cell-derived xenograft tissues. Scale bars=100μm. Quantitation of tumor weight or Ki67+ cells represent means ± SD. (N). Bioluminescence images of lung-colonized tumor cells injected through the tail vein using NOD/SCID mice at the tenth week (n=6 per group). Supplementary file6 (TIF 16075 KB)

Figure S4. Dasatinib treatment inhibits CSC-like properties and cell growth of TNBC. (A-B). Tumorsphere formation ability was analyzed in CAL51 or MDA-MB-231 cells treated with DMSO or Dastinib. Representative images of tumorspheres were shown. Scale bars=100 μm. The quantitation data represent means ± SD with 3 biological replicates. (C-D). Representative images showed the populations of BCSCs (CD44+/CD24-/low) analyzed by flow cytometry in CAL51 or MDA-MB-231 cells treated with DMSO or Dastinib. The quantitation data represent means ± SD with 3 biological replicates. (E-F). Cell viability was measured by CCK-8 assay in CAL51 or MDA-MB-231 cells treated with DMSO or Dastinib. The quantitation data represent means ± SD with 3 biological replicates. Supplementary file7 (TIF 3878 KB)

Figure S5. SRC directly activates YAP1 independently of canonical Hippo kinases. (A-B). The heatmap indicated the gene expression changes induced by overexpression of CA-SRC or SRC knockdown in CAL51 cells analyzed by RNA-seq. (C). The commonly upregulated or downregulated gene numbers identified by RNA-seq in CAL51 cells stably expressing two distinct SRC shRNAs. (D-E). Gene set enrichment analysis on the SRC-regulated transcriptome and the oncogenic signatures enriched in the SRC-regulated oncogenic program. (F). Luciferase assay using empty control (pGL3Ctrl) or TEAD-dependent reporter (8xGTIIC) in 293T cells transiently transduced with empty vector, YAP1, CA-SRC or CA-SRC+YAP1 plasmids respectively. The data are shown as the mean ± SD (n=3). (G). Western blot analyses of total proteins from the MDA-MB-231 cells stably expressing Ctrl, CA-SRC or shSRC using the indicated antibodies. (H-I). Western blot analyses of LATS1 and MOB1 tyrosine phosphorylation level in CAL51 cells stably expressing Ctrl and CA-SRC using p-Tyr antibody after LATS1 or MOB1 immunoprecipitation. (J). Western blot analyses of total proteins from the CAL51 or MDA-MB-231 cells stably expressing Ctrl, CA-SRC or shSRC using the indicated antibodies. (K). Luciferase assay using pGL3Ctrl or TEAD-dependent reporter (8xGTIIC) in 293T cells transiently transduced with empty vector, CA-SRC plasmid or LATS1/2 siRNA respectively. The data are shown as the mean ± SD (n=3). Supplementary file8 (TIF 4162 KB)

Figure S6. Identification of a new YAP1/KLF5 oncogenic module. (A). Western blot analyses of total proteins from the MCF10A cells stably expressing hYAP1 sgRNA and the indicated overexpressing plasmids. (B-C). Cell growth curve and colony-formation analysis of the MCF10A cells form Figure S6A in the cell culture medium without EGF. The data are shown as the mean ± SD (n=3). (D). The flowchart shows the experiment design to identify the YAP1 tyrosine phosphorylation-dependent interacting partners using CA-SRC cells stable transduced with WT-YAP1 or mutant-YAP1. (E). The unique KLF5 peptides identified by LC-MS in Flag-WT-YAP1 immunoprecipitates. (F). The partial list of representative proteins identified by Flag-IP-MS. (G). Western blot analyses of input or Flag-IP proteins from CA-SRC cells stably expressing Ctrl, Flag-WT or mut-YAP1 using the indicated antibodies. (H). Co-IP showing the interaction between exogenous YAP1 and KLF5 in 293T cells, stably expressing Ctrl or CA-SRC plasmids. (I). Luciferase assay using 8xGTIIC reporter in 293T cells transiently transduced with different concentrations of YAP1/KLF5 overexpressing plasmids respectively. The data are shown as the mean ± SD (n=3). (J-K). Luciferase assay using 8xGTIIC reporter in CAL51 cells transiently transduced with YAP1 or KLF5 shRNAs respectively, or WT-YAP1/mut-YAP1+KLF5 overexpressing plasmids. The data are shown as the mean ± SD (n=3). (L-M). The heatmap indicated the gene expression changes induced by knockdown of YAP1 or KLF5 in CAL51 cells analyzed by RNA-seq. The commonly upregulated or downregulated gene numbers were shown. (N). Quantitative real-time PCR to examine the mRNA level of the indicated gene expression in CAL51 cells stably expressing shCtrl or shKLF5. The data are shown as the mean ± SD (n=3). Statistically significant differences were indicated. Supplementary file9 (TIF 5145 KB)

Figure S7. Knockdown of either YPA1 or KLF5 inhibited BC cell metastasis. (A-H). Bioluminescence images of lung-colonized tumor cells injected through the tail vein using NOD/SCID mice at the tenth week (n=6 per group), the quantification data was based on the bioluminescence signal intensities and represent means ± SD. HE staining of sections from lung nodules and the quantification data represent the relative area of lung nodules. n=6. Scale bar=100 µm. Supplementary file10 (TIF 10240 KB)

Figure S8. YAP1-KLF5 oncogenic module is responsible for SRC-enhanced CSC-like properties, cell growth and migration/invasion behaviors. (A-B). Representative images showed the populations of BCSCs (CD44+/CD24-/low) analyzed by flow cytometry in CAL51 cells stably expressing Ctrl, CA-SRC or CA-SRC+ShYAP1 or ShKLF5. The quantitation data represent means ± SD with 3 biological replicates. (C-D). Tumorsphere formation was analyzed in CAL51 or MDA-MB-231 cells stably expressing Ctrl, CA-SRC or CA-SRC+shYAP1 or shKLF5. Representative images of tumorspheres were shown. Scale bars=100 μm. The quantitation data represent means ± SD with 3 biological replicates. (E-F). Cell viability was measured by CCK-8 assay in CAL51 or MDA-MB-231 cells stably expressing Ctrl, CA-SRC or CA-SRC+shYAP1 or shKLF5. The quantitation data represent means ± SD with 3 biological replicates. (G-H). In vitro cell migration/invasion ability was measured in CAL51 cells stably expressing Ctrl, CA-SRC or CA-SRC+shYAP1, or shKLF5 using the Transwell chamber or Transwell chamber containing the Matrigel as barrier. Representative images of migrated cells were shown. Scale bars=100 μm. The quantitation data represent means ± SD with 3 biological replicates. Supplementary file11 (TIF 13577 KB)

Figure S9. Clinical relevance of SRC-YAP1/KLF5 signaling axis with human BCs. (A-B). YAP1 protein expression levels in 6 normal and 74 BC tissues were detected by IHC and analyzed by IHC scores. (C-D). Quantitation of YAP1 expression level in 74 BC tissues with different subtypes or TNM stages according to the IHC scores. (E-F). mRNA and protein expressions of KLF5 BRCA-based sample types. Supplementary file12 (TIF 2217 KB)

Figure S10. Full western blot images for Figure 1-2. Supplementary file13 (TIF 10359 KB)

Figure S11. Full western blot images for Figure 3. Supplementary file14 (TIF 5650 KB)

Figure S12. Full western blot images for Figure 4. Supplementary file15 (TIF 10365 KB)

Figure S13. Full western blot images for Figure 5 and S2. Supplementary file16 (TIF 6931 KB)

Figure S14. Full western blot images for Figure S3 and S5. Supplementary file17 (TIF 9826 KB)

Figure S15. Full western blot images for Figure S6. Supplementary file18 (TIF 4019 KB)

## Data Availability

The datasets used and analyzed in this study are available from the corresponding author on reasonable request.

## Abbreviations

BC: Breast cancer
BCSC: Breast cancer stem cell
Co-IP: Coimmunoprecipitation
DEG: Differentially expressed genes
EMT: Epithelial-mesenchymal transition
GOF: Gain-of-function
IHC: Immunohistochemistry
KLF5: Kruppel like factor 5
LOF: Loss-of-function
SD: Standard deviation
SFK: SRC family kinase
SH: SRC-homology
TCGA: The cancer genome atlas
TMAs: Tissue microarrays
TNBC: Triple-negative breast cancer
TNM: Tumor node metastasis
