# Supplementary material for: Tyrosine kinase SRC-induced YAP1-KLF5 module regulates cancer stemness and metastasis in triple-negative breast cancer
Source: Cell Mol Life Sci. 2023 Jan 12;80(2):41. doi: 10.1007/s00018-023-04688-w (PMC9837006; doi:10.1007/s00018-023-04688-w)
Supplement: Supplementary file 1 — Supplementary file1 (DOCX 20 KB) [file 18_2023_4688_MOESM1_ESM.docx]

**Supplementary table 1: List of oligos used in this study**

| **Target gene** | **Oligo sequences** | **Experiment used** |
| --- | --- | --- |
| *GAPDH* | Forward: 5'-TCGGAGTCAACGGATTTGGT-3'  Reverse: 5'-TTCCCGTTCTCAGCCTTGAC-3' | qRT-PCR |
| *YAP1* | Forward: 5’-ACCTTGAAGCCATTCCTGGG-3’  Reverse: 5’-TCCTGCAGACTTGGCATCAG-3’ | qRT-PCR |
| *SRC* | Forward: 5’-GGACAGACAGGCTACATCCC-3’  Reverse: 5’- TCTGACTCCCGTCTGGTGAT-3’ | qRT-PCR |
| *CTGF* | Forward: 5’-AGGTGTGGCTTTAGGAGCAG-3’  Reverse: 5’-TCTTGATGGCTGGAGAATGC -3’ | qRT-PCR |
| *CYR61* | Forward: 5’-TGGAACTGGTATCTCCACACG-3’  Reverse: 5’-TACACTGGCTGTCCACAAGG -3’ | qRT-PCR |
| *CD24* | Forward: 5’- TGCTCCTACCCACGCAGA -3’  Reverse: 5’- TGAGACCACGAAGAGACTGG-3’ | qRT-PCR |
| *CD44* | Forward: 5’- CCCATCCCAGACGAAGACAG -3’  Reverse: 5’- CCAATCCCAGGTTTCTTGCCT -3’ | qRT-PCR |
| *SPP1* | Forward: 5’- ATAGAGCTGCCTTGGGGGTC -3’  Reverse: 5’- GCCACAGCATCTGGGTATTTG -3’’ | qRT-PCR |
| *DLL1* | Forward: 5’- ACCGCTATGTGTGCGAGTGT -3’  Reverse: 5’- TGAGGACAAGGATGACCCCG -3’ | qRT-PCR |
| *IL1B* | Forward: 5’- CTCGCCAGTGAAATGATGGCT -3’  Reverse: 5’- TGGTGGTCGGAGATTCGTAG -3’ | qRT-PCR |
| *GAS6* | Forward: 5’- AACCTGACCGTGGGAGGTAT -3’  Reverse: 5’- TTCTCCGTTCAGCCAGTTCC -3’ | qRT-PCR |
| *GLS* | Forward: 5’- AAAAGCAGTCTGGAGGAAAGGT-3’  Reverse: 5’ -CCCCACAAATCGGGACTGAA -3’ | qRT-PCR |
| *ITGB2* | Forward: 5’-GGCGTGTTGAGTGTAGTGGT -3’  Reverse: 5’-CGCTGCAGTTCTTCCCAAAG -3’ | qRT-PCR |
| *TAGLN* | Forward: 5’- GGCTGGTGGAGTGGATCATA-3’  Reverse: 5’-TTGGAGCCATCAGGGTACAG -3’ | qRT-PCR |
| *KLF5* | Forward: 5’- ATGCCCCCTTGCACATACAC -3’  Reverse: 5’- TGGTGACGGGGGAAAGTAAG -3’ | qRT-PCR |
| *CYR61-1* | Forward: 5’- TAACCTGTGTACGTGTTGGG -3’  Reverse: 5’- GGGGTGGGAGACTTTTCAAGA -3’ | ChIP-qPCR |
| *CYR61-2* | Forward: 5’- CGCCAACCAGCATTCCTGA -3’  Reverse: 5’- CTTTTATACGGGCCGGCGGA -3’ | ChIP-qPCR |
| *CTGF-1* | Forward: 5’- ATATGAATCAGGAGTGGTGCGA -3’  Reverse: 5’- CAACTCACACCGGATTGATCC -3’ | ChIP-qPCR |
| *CTGF-2* | Forward: 5’- TGTGCCAGCTTTTTCAGACG -3’  Reverse: 5’- TGAGCTGAATGGAGTCCTACACA -3’ | ChIP-qPCR |
| *KLF5-1* | Forward: 5’- GAACTTCAAACTCCCCTTTGT -3’  Reverse: 5’- CCAAGCTGCTAGATTTTCAGGA -3’ | ChIP-qPCR |
| *KLF5-2* | Forward: 5’- GGCATGAAGAACTCACATTGCT -3’  Reverse: 5’- TGGGGAACACCCTGTGAATG-3’ | ChIP-qPCR |
| hSRC-shRNA-1 | CCGGGCTCGGCTCATTGAAGACAATCTCGAGATTGTCTTCAATGAGCCGAGCTTTTT | SRC knockdown |
| hSRC-shRNA-2 | CCGGGACAGACCTGTCCTTCAAGAACTCGAGTTCTTGAAGGACAGGTCTGTCTTTTT | SRC knockdown |
| hYAP1-shRNA-1 | CCGGGCCACCAAGCTAGATAAAGAACTCGAGTTCTTTATCTAGCTTGGTGGCTTTTT | YAP1 knockdown |
| hYAP1-shRNA-2 | CCGGGACCAATAGCTCAGATCCTTTCTCGAGAAAGGATCTGAGCTATTGGTCTTTTT | YAP1 knockdown |
| hKLF5-shRNA-1 | CCGGCCTATAATTCCAGAGCATAAACTCGAGTTTATGCTCTGGAATTATAGGTTTTT | KLF5 knockdown |
| hKLF5-shRNA-2 | CCGGCCTATAATTCCAGAGCATAAACTCGAGTTTATGCTCTGGAATTATAGGTTTTTG | KLF5 knockdown |
