# Supplementary material for: Tyrosine kinase SRC-induced YAP1-KLF5 module regulates cancer stemness and metastasis in triple-negative breast cancer
Source: Cell Mol Life Sci. 2023 Jan 12;80(2):41. doi: 10.1007/s00018-023-04688-w (PMC9837006; doi:10.1007/s00018-023-04688-w)
Supplement: Supplementary file 2 — Supplementary file2 (DOCX 24 KB) [file 18_2023_4688_MOESM2_ESM.docx]

**Supplementary table 2: List of antibodies used in this study**

| **Target protein** | **Description** | **Company**  **Cat no.** | **Working concentration** | **Experiment used** |
| --- | --- | --- | --- | --- |
| SRC | [KO Validated] SRC rabbit mAb | A19119 | 1:1000 | WB/IHC |
| pY416SRC | Phospho-SRC family (Tyr416) antibody | CST #2101 | 1:1000 | WB |
| YAP1 | [KO Validated] YAP1 | ABclonal A1002 | 1:3000 | WB/IHC |
| pS127YAP1 | Phospho-YAP1-S127 rabbit pAb | CST #4911 | 1:1000 | WB |
| pS397YAP1 | Phospho-YAP1-S397 (D1E7Y) rabbit pAb | CST #13619 | 1:1000 | WB |
| pY357YAP1 | Anti-YAP1 (Phospho Y357) | Ab62751 | 1:1000 | WB |
| Flag | Mouse anti DDDDK-Tag mAb | ABclonal AE005 | 1:3000 | WB |
| β-Actin | ACTB monoclonal antibody | ABclonal AC004 | 1:5000 | WB |
| C-MYC | c-Myc rabbit mAb | ABclonal A19032 | 1:1000 | WB |
| E-Cadherin | E-Cadherin rabbit pAb | ABclonal A11492 | 1:1000 | WB |
| Vimentin | Vimentin rabbit pAb | ABclonal A11952 | 1:2000 | WB |
| Snail | Snail rabbit pAb | ABclonal A5243 | 1:1000 | WB |
| SOX2 | [KO Validated] SOX2 rabbit pAb | ABclonal A0561 | 1:3000 | WB |
| OCT4 | Oct-3/4 (C-10) mouse monoclonal antibody | Santa cruz sc-5279 | 1:500 | WB |
| NANOG | Anti-NANOG rabbit polyclonal antibody | Sangon Biotech D155241 | 1:500 | WB |
| CTGF | CTGF rabbit polyclonal antibody | Proteintech 23936-1-AP | 1:1000 | WB |
| CYR61 | CYR61 rabbit pAb | ABclonal  A1111 | 1:1000 | WB |
| KLF5 | KLF5 polyclonal rabbit antibody | GeneTex GTX103289 | 1:1000 | WB/IHC |
| Myc-tag | Rabbit anti Myc-Tag pAb-C-terminal | ABclonal AE009 | 1:5000 | WB/IP |
| HA-tag | Mouse anti HA-Tag mAb | ABclonal AE008 | 1:5000 | WB |
| LATS1 | LATS1 Rabbit mAb | ABclonal  A22287 | 1:1000 | WB |
| pLATS1 | Phospho-LATS1 Rabbit mAb | CST 8654 | 1:500 | WB |
| MOB1 | MOB1A Polyclonal antibody | Proteintech  12790-1-AP | 1:1000 | WB |
| p-Tyr | Anti-Phospho-Tyrosine Rabbit mAb | PTMBIO PTM-702RM | 1:500 | WB |
| AKT | [KO Validated] Akt1 Rabbit mAb | Abclonal  A17909 | 1:2000 | WB |
| pAKT | Phospho-Akt1-S473 Rabbit mAb | ABclonal  AP0637 | 1:1000 | WB |
| ERK | ERK1 / ERK2 Rabbit pAb | ABclonal  A16686 | 1:2000 | WB |
| pERK | Phospho-ERK1-T202/Y204 + ERK2-T185/Y187 Rabbit pAb | ABclonal  AP0472 | 1:1000 | WB |
| YAP1 | YAP1 Antibody | Novus  NB110-58358 | 2-10μg | ChIP |
| TEAD4 | TEF-3 Antibody (N-G2) | Santa Cruz  sc-101184 | 2-10μg | ChIP |
| KLF5 | KLF5 polyclonal rabbit antibody | GeneTex GTX103289 | 2-10μg | ChIP |
| Anti-Rabbit | HRP goat anti-rabbit IgG (H+L) | ABclonal AS014 | 1:5000 | WB |
| Anti-Mouse | HRP goat anti-mouse IgG (H+L) | ABclonal AS003 | 1:5000 | WB |
| Anti-Rabbit | HRP-conjugated affinipure rabbit anti-rabbit IgG light chain | ABclonal AS061 | 1:5000 | WB |
| Anti-Mouse | HRP-conjugated affiniPure goat anti-mouse IgG light chain | ABclonal AS062 | 1:5000 | WB |
| Rabbit control IgG | Rabbit control IgG | ABclonal AC005 | 1:100 | IP |
