# Supplementary figures and images for: Tyrosine kinase SRC-induced YAP1-KLF5 module regulates cancer stemness and metastasis in triple-negative breast cancer
Source: Cell Mol Life Sci. 2023 Jan 12;80(2):41. doi: 10.1007/s00018-023-04688-w (PMC9837006; doi:10.1007/s00018-023-04688-w)

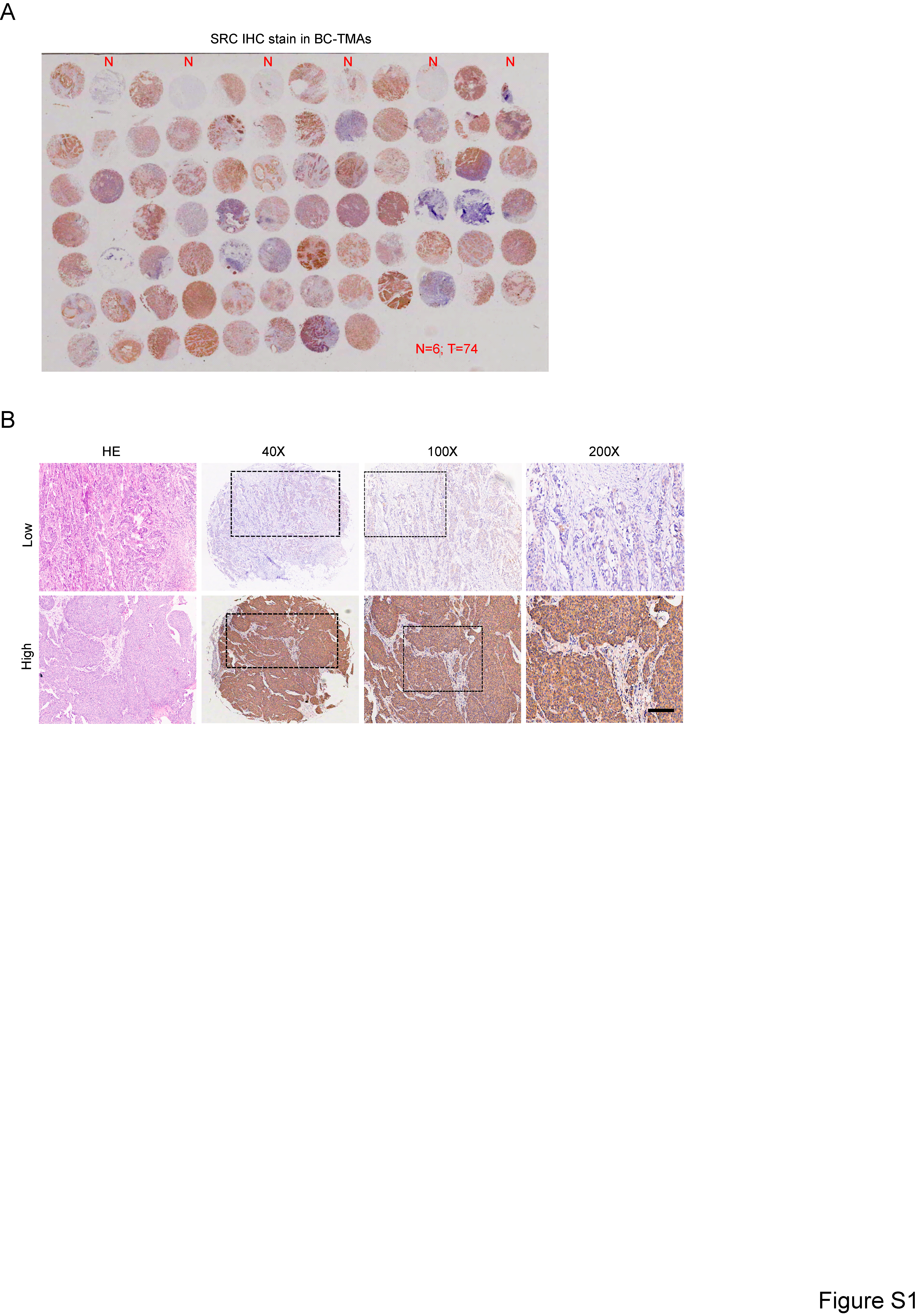

Supplement: Supplementary file 4 — Figure S1. SRC expression in human BC tissues and cells. (A). The whole slide scans for SRC IHC stain with 6 normal tissues and 74 BC tissues. 6 normal tissues have been indicated as “N” in the figure and all the others are BC tissues indicated with “T”. (B). Representative images indicated the low (score ≤6) and high (score >6) expression level of SRC in BC tissues detected with IHC. Scale bars=100 μm. Supplementary file4 (TIF 16393 KB) [file 18_2023_4688_MOESM4_ESM.tif]

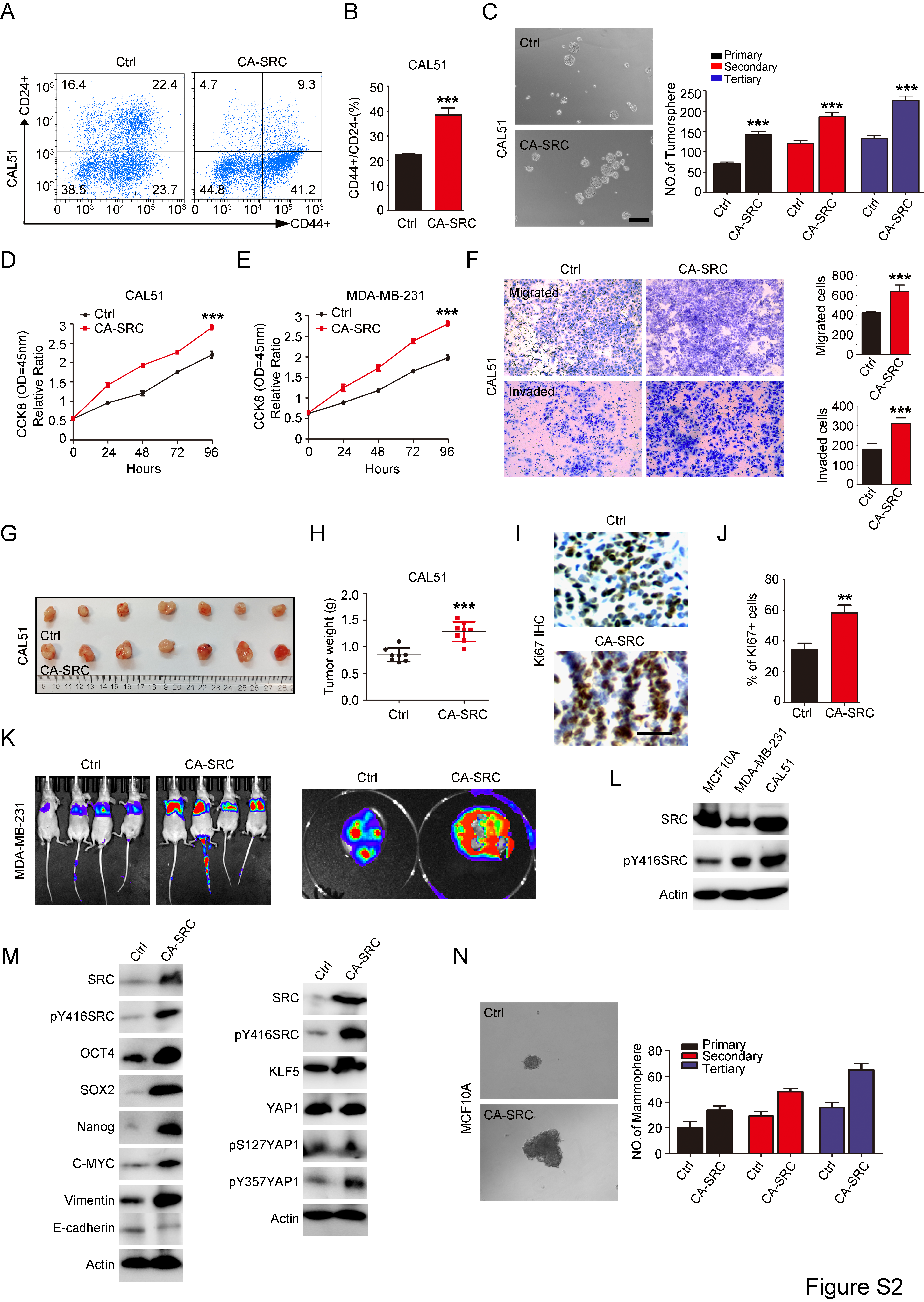

Supplement: Supplementary file 5 — Figure S2. SRC-GOF enhances CSC-like properties, cell growth and migration/invasion behaviors. (A-B). Representative images showed the populations of BCSCs (CD44+/CD24-/low) analyzed by flow cytometry in CAL51 cells stably expressing Ctrl or CA-SRC. The quantitation data represent means ± SD with 3 biological replicates. (C). Tumorsphere formation ability was analyzed in Ctrl or CA-SRC CAL51 cells. Representative images of tumorspheres were shown. Scale bars=100 μm. The quantitation data represent means ± SD with 3 biological replicates. (D-E). Cell viability was measured by CCK-8 assay in CAL51 or MDA-MB-231 cells stably expressing Ctrl or CA-SRC. The quantitation data represent means ± SD with 3 biological replicates. (F). In vitro cell migration/invasion ability was measured in Ctrl or CA-SRC CAL51 cells using the Transwell chamber or Transwell chamber containing the Matrigel as barrier. Representative images of migrated/invaded cells were shown. Scale bars=100 μm. The quantitation data represent means ± SD with 3 biological replicates. (G-J). Xenograft tumor formation assays in NOD-SCID mice using CAL51 cells stably expressing Ctrl or CA-SRC. IHC analyses of Ki67 protein expression in CAL51 cell-derived xenograft tissues. Scale bars=100 μm. Quantitation of tumor weight or Ki67+ cells represent means ± SD. (K). Bioluminescence images of lung-colonized tumor cells injected through the tail vein using NOD/SCID mice at the tenth week (n=6 per group). (L-M). Western blot analyses of total proteins from the MCF10A cells stably expressing Ctrl or CA-SRC using the indicated antibodies. (N). Mammosphere formation efficiency was determined in Ctrl or CA-SRC MCF10A cells. Representative images of mammospheres were shown. Scale bars=100 μm. The quantitation data represent means ± SD with 3 biological replicates. Supplementary file5 (TIF 14957 KB) [file 18_2023_4688_MOESM5_ESM.tif]

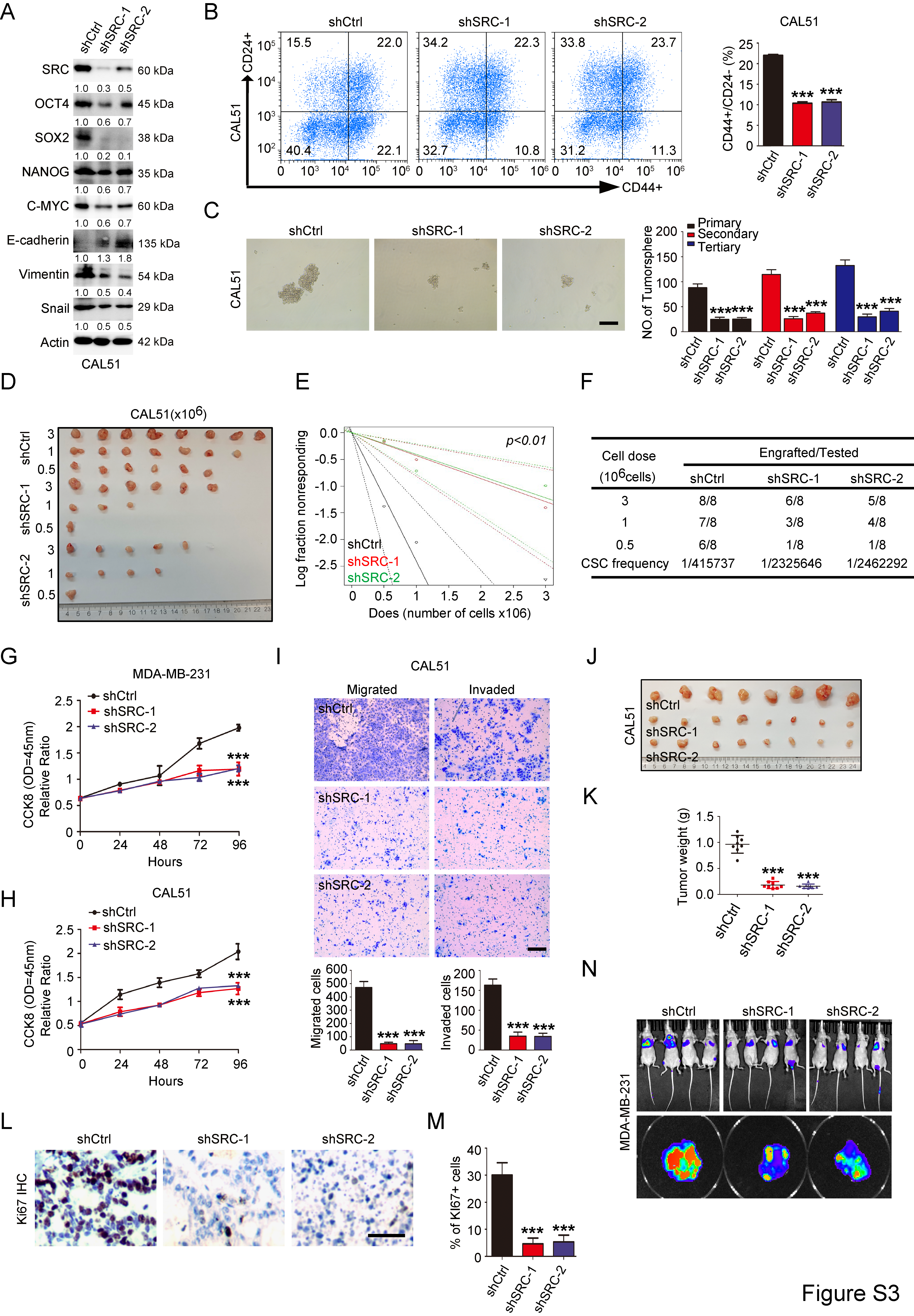

Supplement: Supplementary file 6 — Figure S3. SRC-LOF reduces CSC-like properties, cell growth and migration/invasion behaviors. (A). Western blot analyses of total proteins from the CAL51 cells stably expressing Ctrl or CA-SRC using the indicated antibodies. The western-blot band intensities of various markers were normalized to the corresponding Actin intensity, and the quantitative values have been provided. (B). Representative images showed the populations of BCSCs (CD44+/CD24-/low) analyzed by flow cytometry in CAL51 cells stably expressing shCtrl or shSRC. The quantitation data represent means ± SD with 3 biological replicates. (C). Tumorsphere formation ability was analyzed in shCtrl or shSRC TNBC cells. Representative images of tumorspheres were shown. Scale bars=100 μm. The quantitation data represent means ± SD with 3 biological replicates. (D-F). Tumor-initiating cell frequency was analyzed by in vivo limiting dilution assay using CAL51 cells stably expressing shCtrl or shSRC, n=8. (G-H). Cell viability was measured by CCK-8 assay in CAL51 or MDA-MB-231 cells stably expressing shCtrl or shSRC. The quantitation data represent means ± SD with 3 biological replicates. (I). In vitro cell migration/invasion ability was measured in shCtrl or shSRC CAL51 cells using the Transwell chamber or Transwell chamber containing the Matrigel as barrier. Representative images of migrated/invaded cells were shown. Scale bars=100 μm. The quantitation data represent means ± SD with 3 biological replicates. (J-M). Xenograft tumor formation assays in NOD-SCID mice using CAL51 cells stably expressing shCtrl or shSRC. IHC analyses of Ki67 protein expression in CAL51 cell-derived xenograft tissues. Scale bars=100μm. Quantitation of tumor weight or Ki67+ cells represent means ± SD. (N). Bioluminescence images of lung-colonized tumor cells injected through the tail vein using NOD/SCID mice at the tenth week (n=6 per group). Supplementary file6 (TIF 16075 KB) [file 18_2023_4688_MOESM6_ESM.tif]

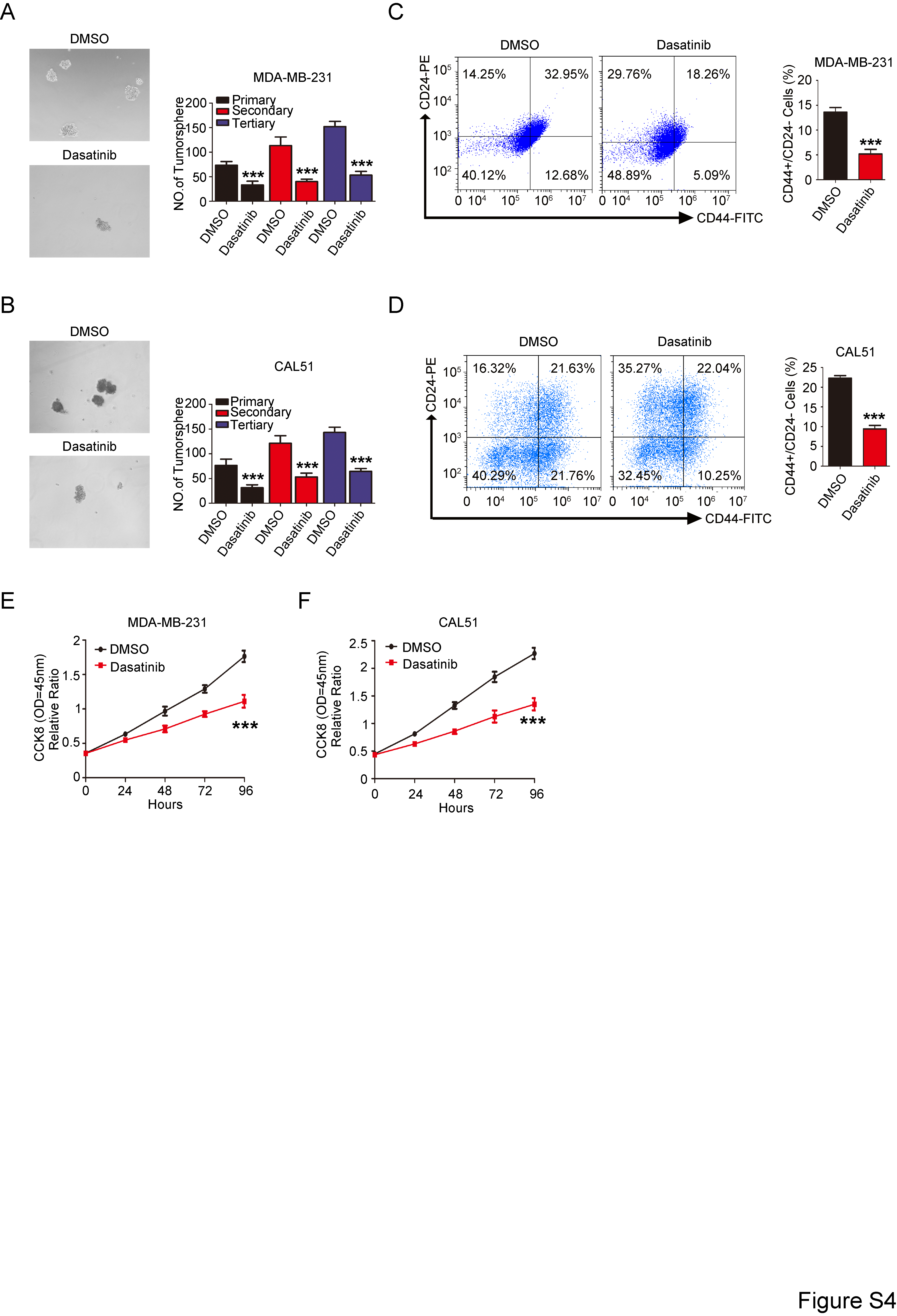

Supplement: Supplementary file 7 — Figure S4. Dasatinib treatment inhibits CSC-like properties and cell growth of TNBC. (A-B). Tumorsphere formation ability was analyzed in CAL51 or MDA-MB-231 cells treated with DMSO or Dastinib. Representative images of tumorspheres were shown. Scale bars=100 μm. The quantitation data represent means ± SD with 3 biological replicates. (C-D). Representative images showed the populations of BCSCs (CD44+/CD24-/low) analyzed by flow cytometry in CAL51 or MDA-MB-231 cells treated with DMSO or Dastinib. The quantitation data represent means ± SD with 3 biological replicates. (E-F). Cell viability was measured by CCK-8 assay in CAL51 or MDA-MB-231 cells treated with DMSO or Dastinib. The quantitation data represent means ± SD with 3 biological replicates. Supplementary file7 (TIF 3878 KB) [file 18_2023_4688_MOESM7_ESM.tif]

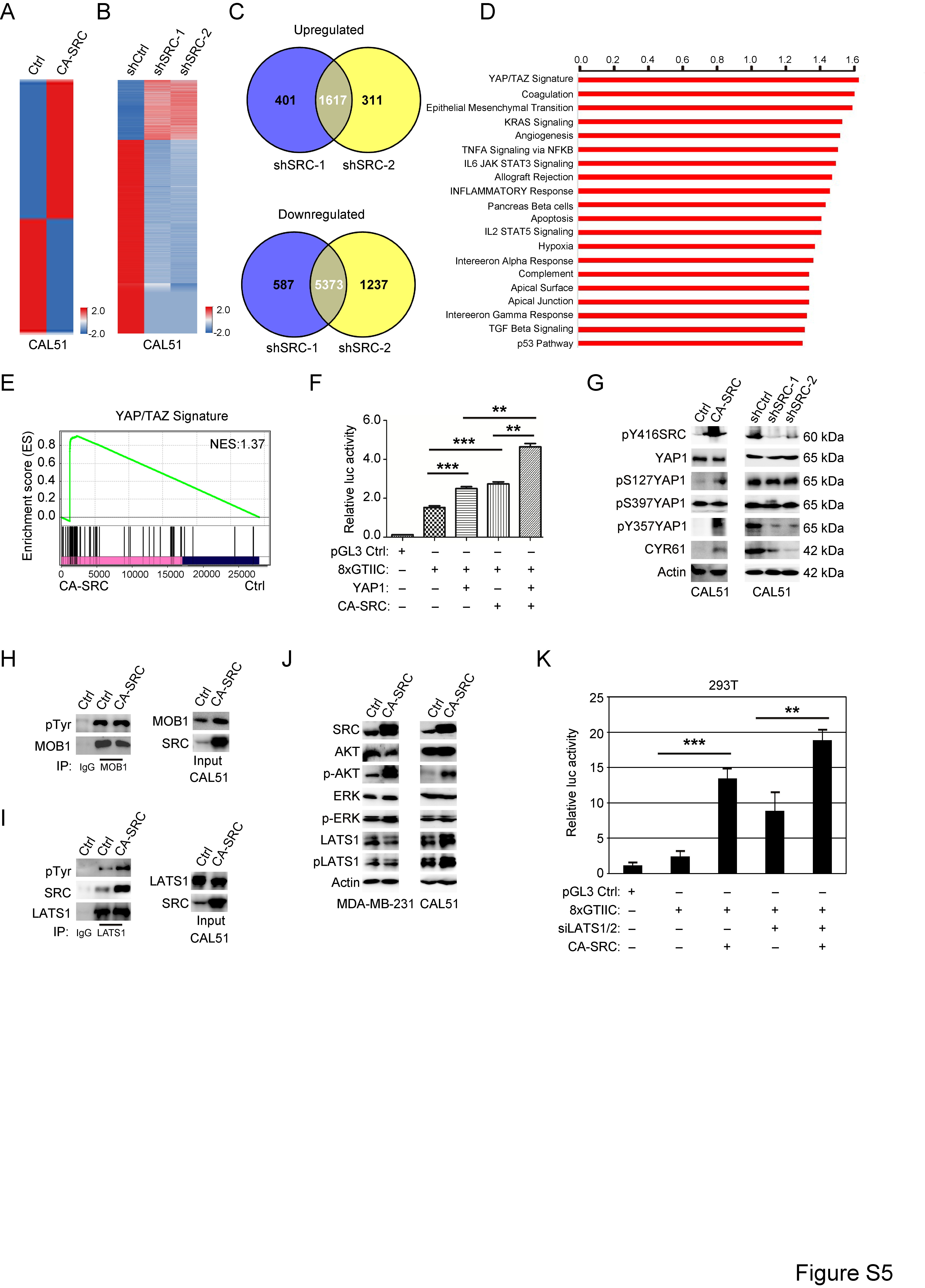

Supplement: Supplementary file 8 — Figure S5. SRC directly activates YAP1 independently of canonical Hippo kinases. (A-B). The heatmap indicated the gene expression changes induced by overexpression of CA-SRC or SRC knockdown in CAL51 cells analyzed by RNA-seq. (C). The commonly upregulated or downregulated gene numbers identified by RNA-seq in CAL51 cells stably expressing two distinct SRC shRNAs. (D-E). Gene set enrichment analysis on the SRC-regulated transcriptome and the oncogenic signatures enriched in the SRC-regulated oncogenic program. (F). Luciferase assay using empty control (pGL3Ctrl) or TEAD-dependent reporter (8xGTIIC) in 293T cells transiently transduced with empty vector, YAP1, CA-SRC or CA-SRC+YAP1 plasmids respectively. The data are shown as the mean ± SD (n=3). (G). Western blot analyses of total proteins from the MDA-MB-231 cells stably expressing Ctrl, CA-SRC or shSRC using the indicated antibodies. (H-I). Western blot analyses of LATS1 and MOB1 tyrosine phosphorylation level in CAL51 cells stably expressing Ctrl and CA-SRC using p-Tyr antibody after LATS1 or MOB1 immunoprecipitation. (J). Western blot analyses of total proteins from the CAL51 or MDA-MB-231 cells stably expressing Ctrl, CA-SRC or shSRC using the indicated antibodies. (K). Luciferase assay using pGL3Ctrl or TEAD-dependent reporter (8xGTIIC) in 293T cells transiently transduced with empty vector, CA-SRC plasmid or LATS1/2 siRNA respectively. The data are shown as the mean ± SD (n=3). Supplementary file8 (TIF 4162 KB) [file 18_2023_4688_MOESM8_ESM.tif]

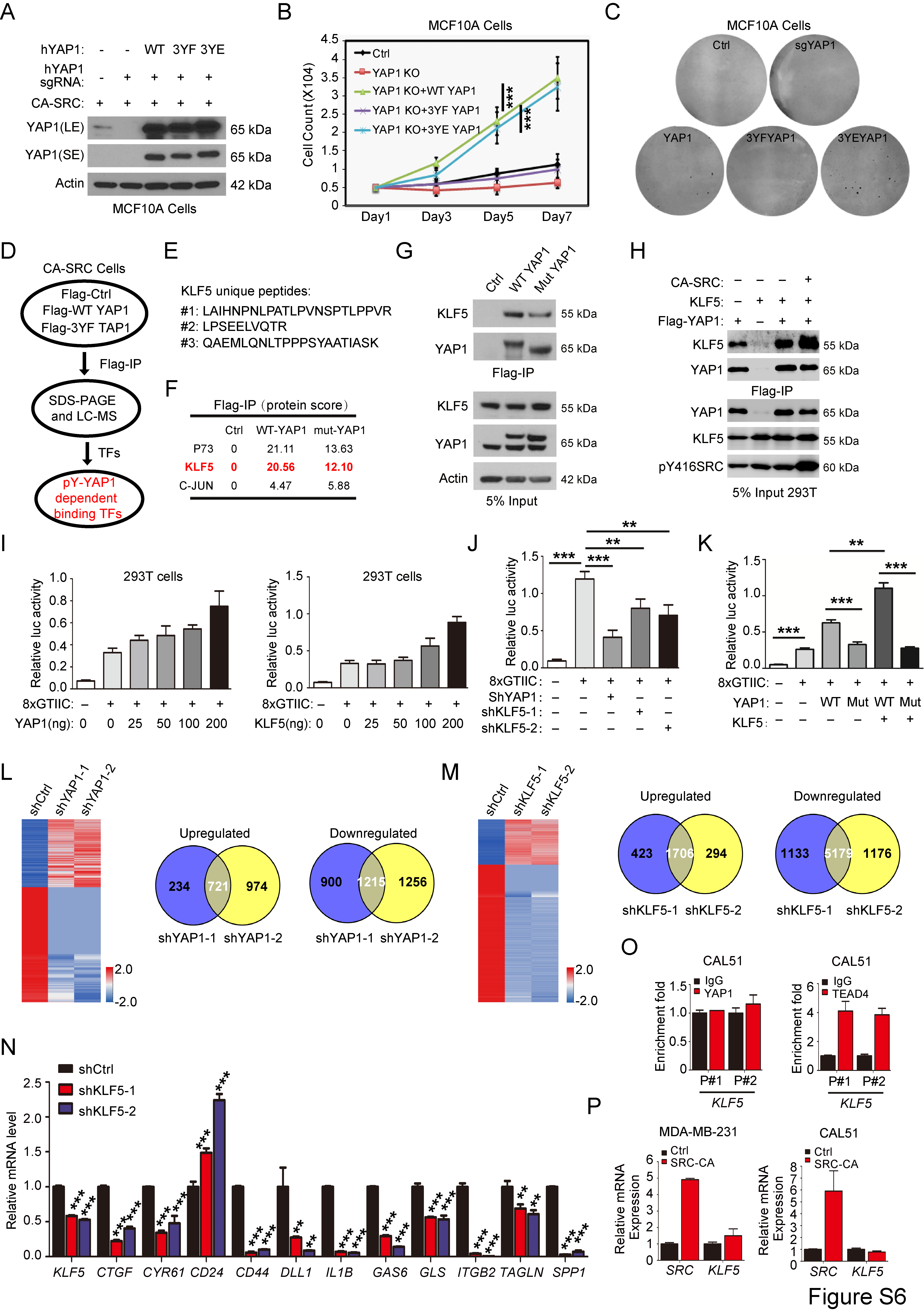

Supplement: Supplementary file 9 — Figure S6. Identification of a new YAP1/KLF5 oncogenic module. (A). Western blot analyses of total proteins from the MCF10A cells stably expressing hYAP1 sgRNA and the indicated overexpressing plasmids. (B-C). Cell growth curve and colony-formation analysis of the MCF10A cells form Figure S6A in the cell culture medium without EGF. The data are shown as the mean ± SD (n=3). (D). The flowchart shows the experiment design to identify the YAP1 tyrosine phosphorylation-dependent interacting partners using CA-SRC cells stable transduced with WT-YAP1 or mutant-YAP1. (E). The unique KLF5 peptides identified by LC-MS in Flag-WT-YAP1 immunoprecipitates. (F). The partial list of representative proteins identified by Flag-IP-MS. (G). Western blot analyses of input or Flag-IP proteins from CA-SRC cells stably expressing Ctrl, Flag-WT or mut-YAP1 using the indicated antibodies. (H). Co-IP showing the interaction between exogenous YAP1 and KLF5 in 293T cells, stably expressing Ctrl or CA-SRC plasmids. (I). Luciferase assay using 8xGTIIC reporter in 293T cells transiently transduced with different concentrations of YAP1/KLF5 overexpressing plasmids respectively. The data are shown as the mean ± SD (n=3). (J-K). Luciferase assay using 8xGTIIC reporter in CAL51 cells transiently transduced with YAP1 or KLF5 shRNAs respectively, or WT-YAP1/mut-YAP1+KLF5 overexpressing plasmids. The data are shown as the mean ± SD (n=3). (L-M). The heatmap indicated the gene expression changes induced by knockdown of YAP1 or KLF5 in CAL51 cells analyzed by RNA-seq. The commonly upregulated or downregulated gene numbers were shown. (N). Quantitative real-time PCR to examine the mRNA level of the indicated gene expression in CAL51 cells stably expressing shCtrl or shKLF5. The data are shown as the mean ± SD (n=3). Statistically significant differences were indicated. Supplementary file9 (TIF 5145 KB) [file 18_2023_4688_MOESM9_ESM.tif]

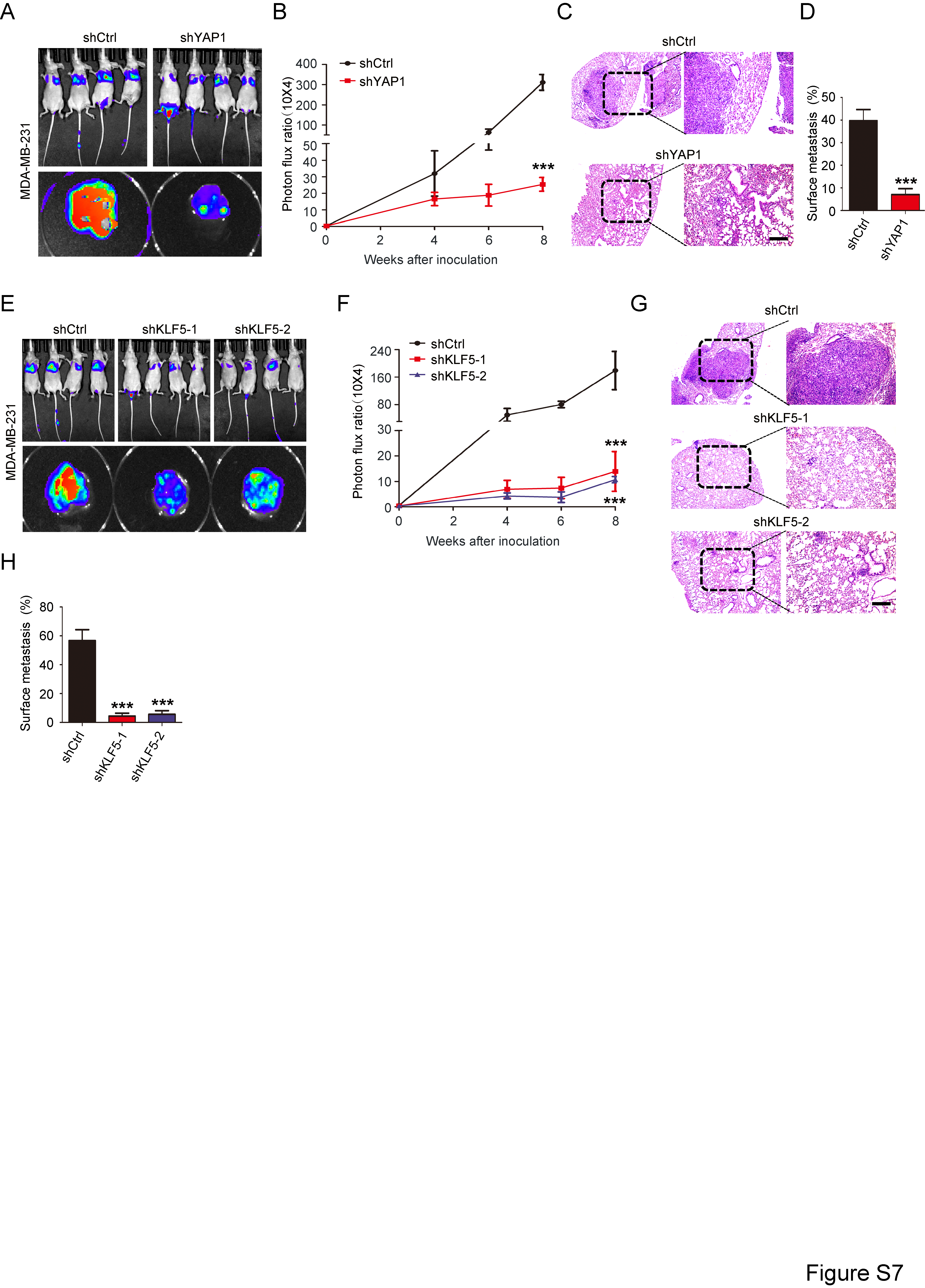

Supplement: Supplementary file 10 — Figure S7. Knockdown of either YPA1 or KLF5 inhibited BC cell metastasis. (A-H). Bioluminescence images of lung-colonized tumor cells injected through the tail vein using NOD/SCID mice at the tenth week (n=6 per group), the quantification data was based on the bioluminescence signal intensities and represent means ± SD. HE staining of sections from lung nodules and the quantification data represent the relative area of lung nodules. n=6. Scale bar=100 µm. Supplementary file10 (TIF 10240 KB) [file 18_2023_4688_MOESM10_ESM.tif]

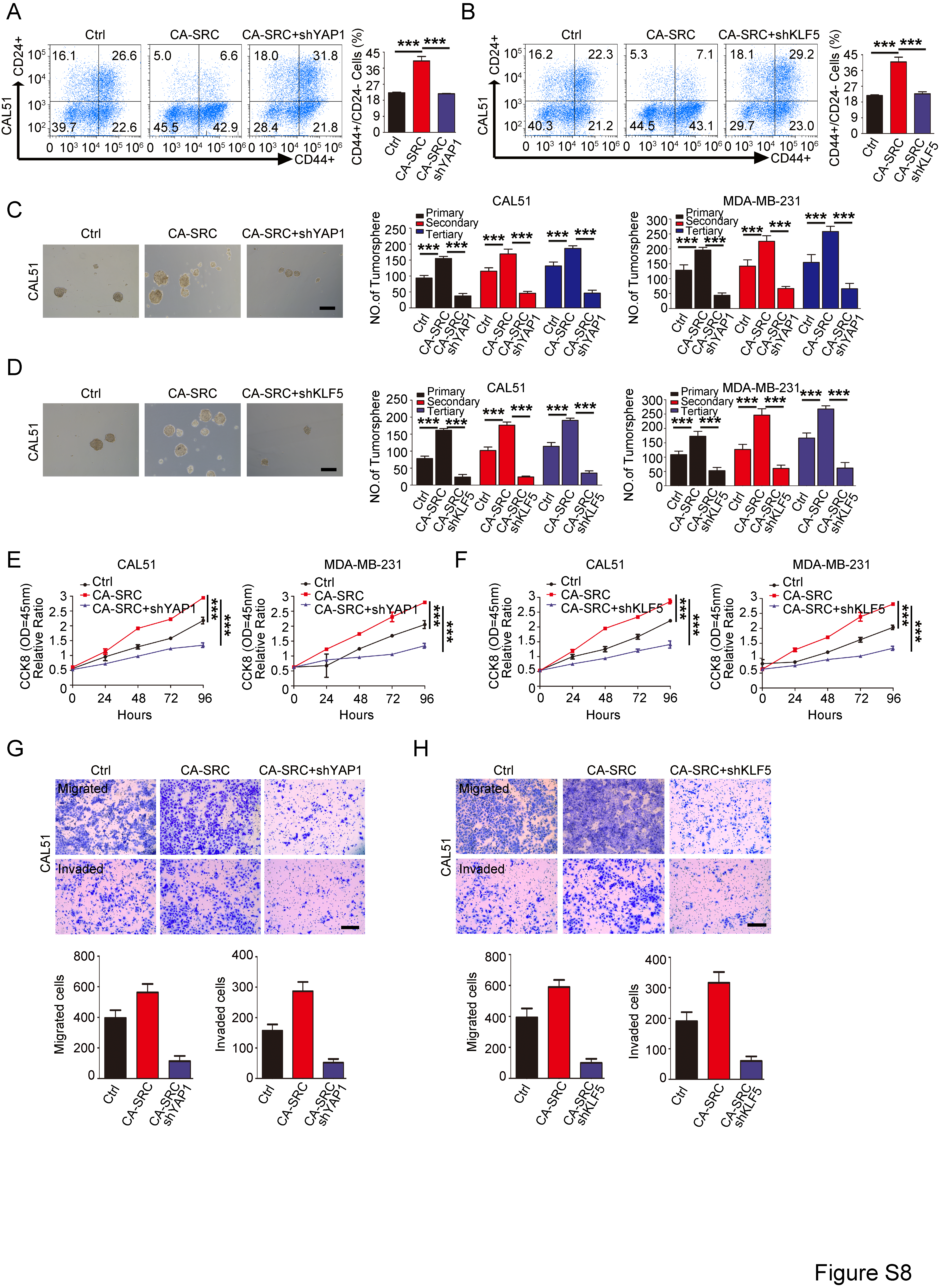

Supplement: Supplementary file 11 — Figure S8. YAP1-KLF5 oncogenic module is responsible for SRC-enhanced CSC-like properties, cell growth and migration/invasion behaviors. (A-B). Representative images showed the populations of BCSCs (CD44+/CD24-/low) analyzed by flow cytometry in CAL51 cells stably expressing Ctrl, CA-SRC or CA-SRC+ShYAP1 or ShKLF5. The quantitation data represent means ± SD with 3 biological replicates. (C-D). Tumorsphere formation was analyzed in CAL51 or MDA-MB-231 cells stably expressing Ctrl, CA-SRC or CA-SRC+shYAP1 or shKLF5. Representative images of tumorspheres were shown. Scale bars=100 μm. The quantitation data represent means ± SD with 3 biological replicates. (E-F). Cell viability was measured by CCK-8 assay in CAL51 or MDA-MB-231 cells stably expressing Ctrl, CA-SRC or CA-SRC+shYAP1 or shKLF5. The quantitation data represent means ± SD with 3 biological replicates. (G-H). In vitro cell migration/invasion ability was measured in CAL51 cells stably expressing Ctrl, CA-SRC or CA-SRC+shYAP1, or shKLF5 using the Transwell chamber or Transwell chamber containing the Matrigel as barrier. Representative images of migrated cells were shown. Scale bars=100 μm. The quantitation data represent means ± SD with 3 biological replicates. Supplementary file11 (TIF 13577 KB) [file 18_2023_4688_MOESM11_ESM.tif]

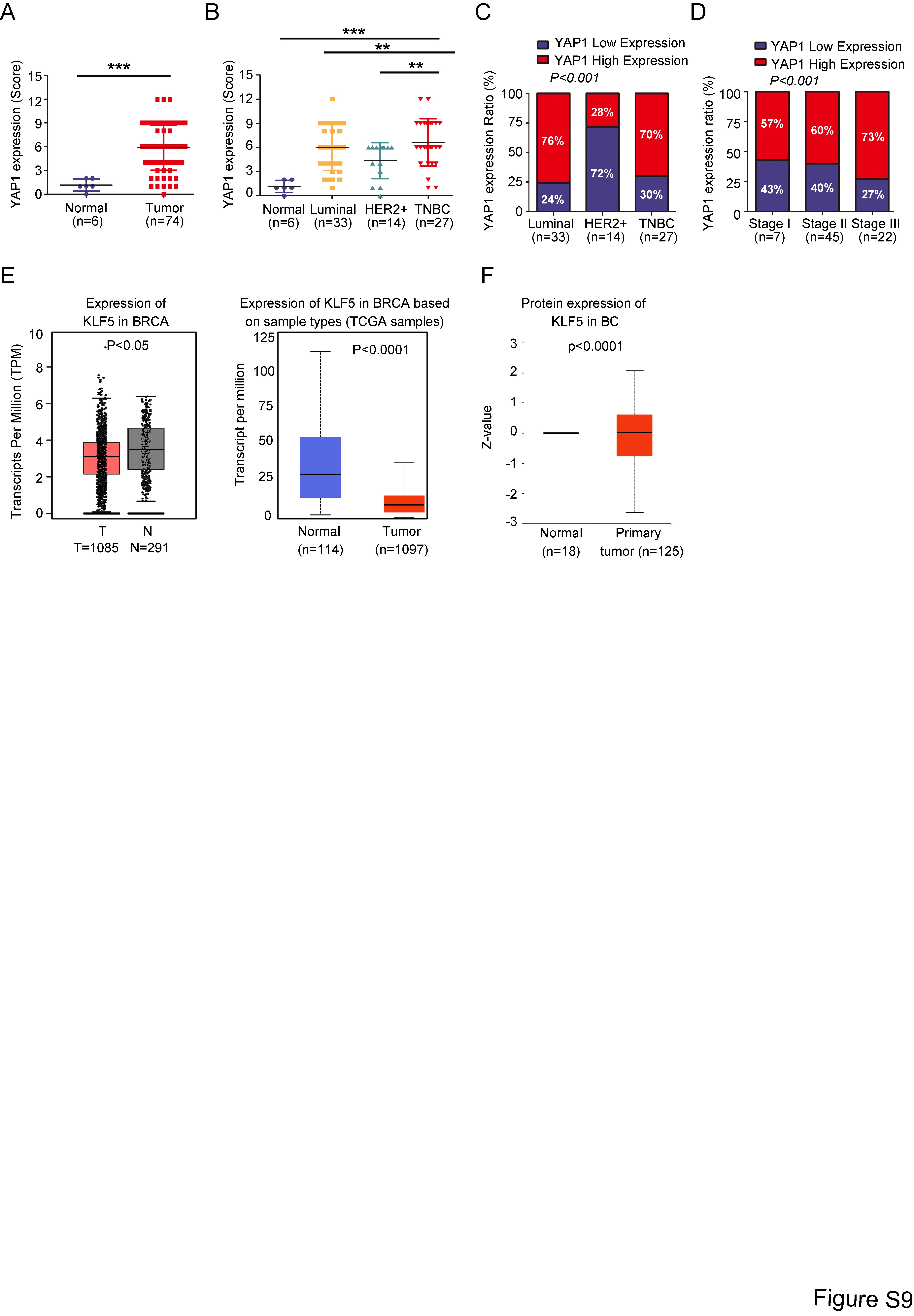

Supplement: Supplementary file 12 — Figure S9. Clinical relevance of SRC-YAP1/KLF5 signaling axis with human BCs. (A-B). YAP1 protein expression levels in 6 normal and 74 BC tissues were detected by IHC and analyzed by IHC scores. (C-D). Quantitation of YAP1 expression level in 74 BC tissues with different subtypes or TNM stages according to the IHC scores. (E-F). mRNA and protein expressions of KLF5 BRCA-based sample types. Supplementary file12 (TIF 2217 KB) [file 18_2023_4688_MOESM12_ESM.tif]

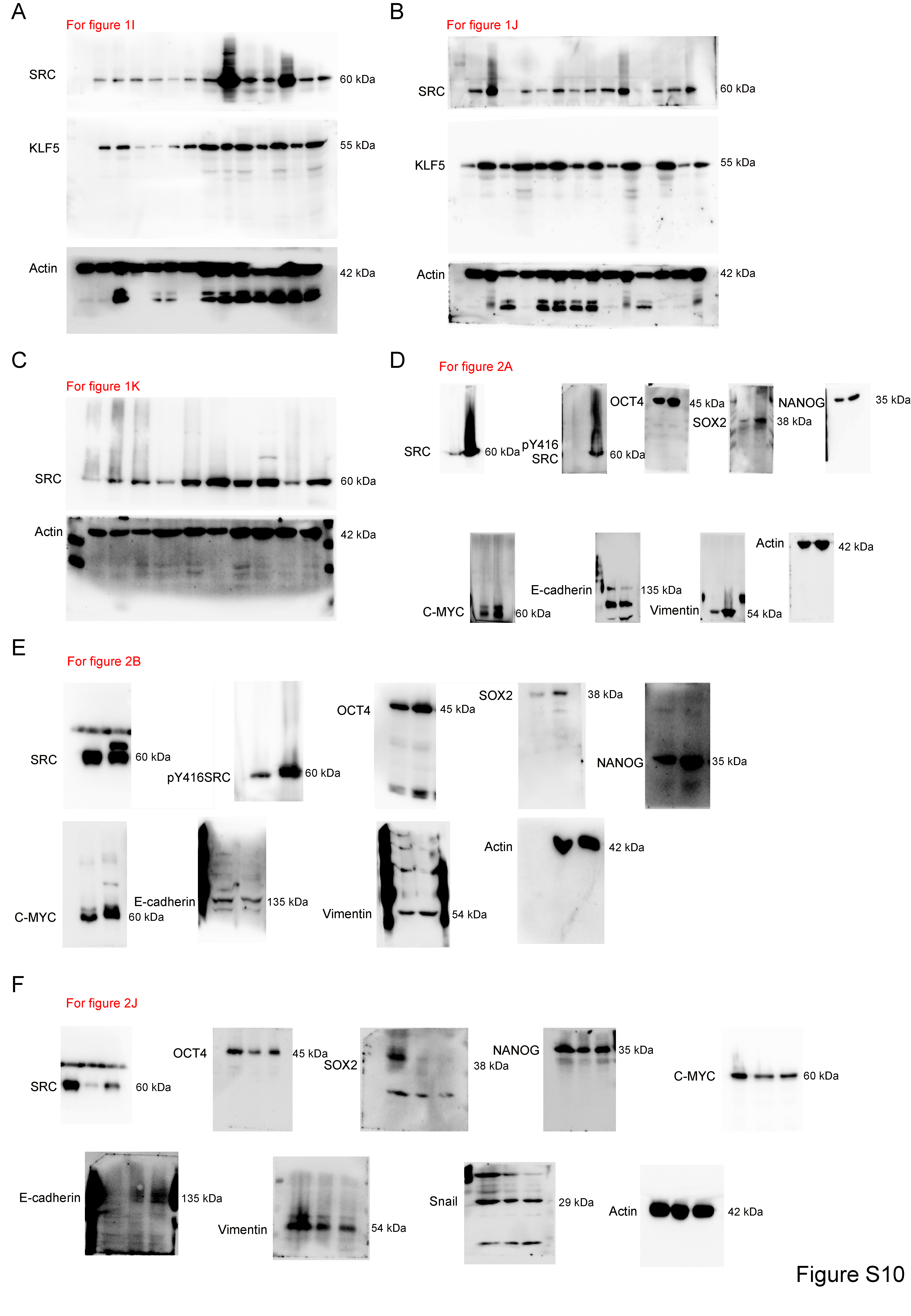

Supplement: Supplementary file 13 — Figure S10. Full western blot images for Figure 1-2. Supplementary file13 (TIF 10359 KB) [file 18_2023_4688_MOESM13_ESM.tif]

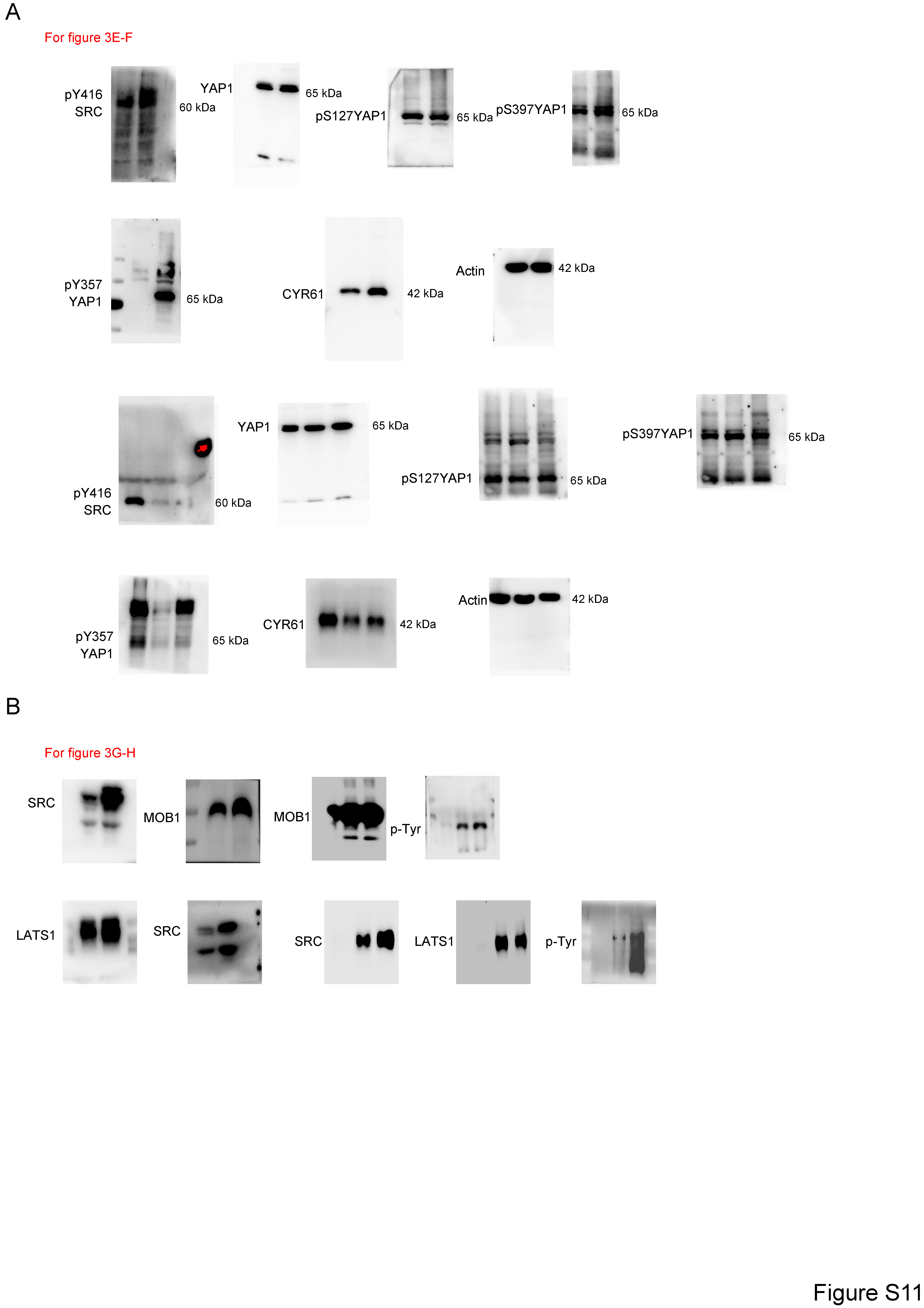

Supplement: Supplementary file 14 — Figure S11. Full western blot images for Figure 3. Supplementary file14 (TIF 5650 KB) [file 18_2023_4688_MOESM14_ESM.tif]

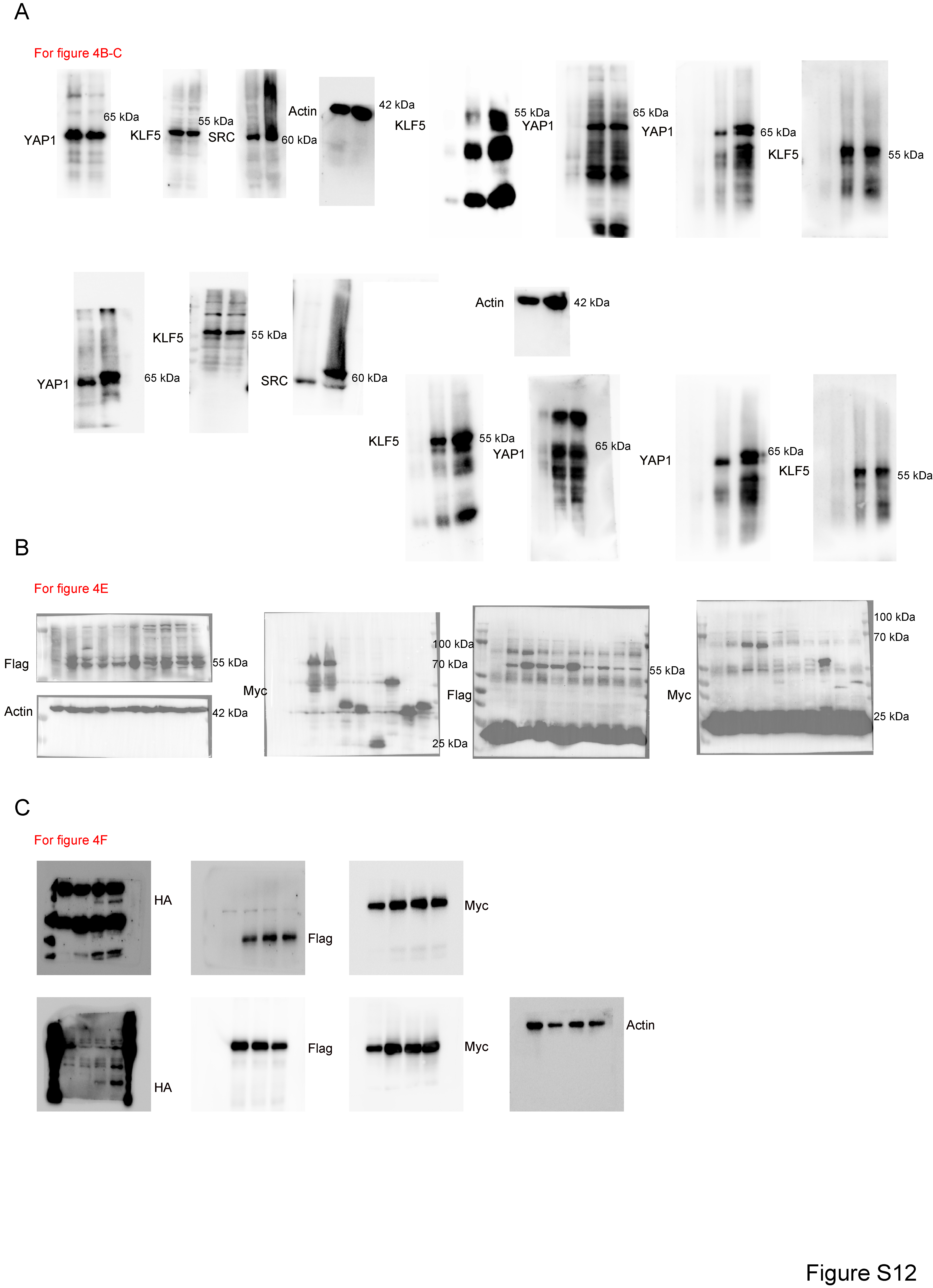

Supplement: Supplementary file 15 — Figure S12. Full western blot images for Figure 4. Supplementary file15 (TIF 10365 KB) [file 18_2023_4688_MOESM15_ESM.tif]

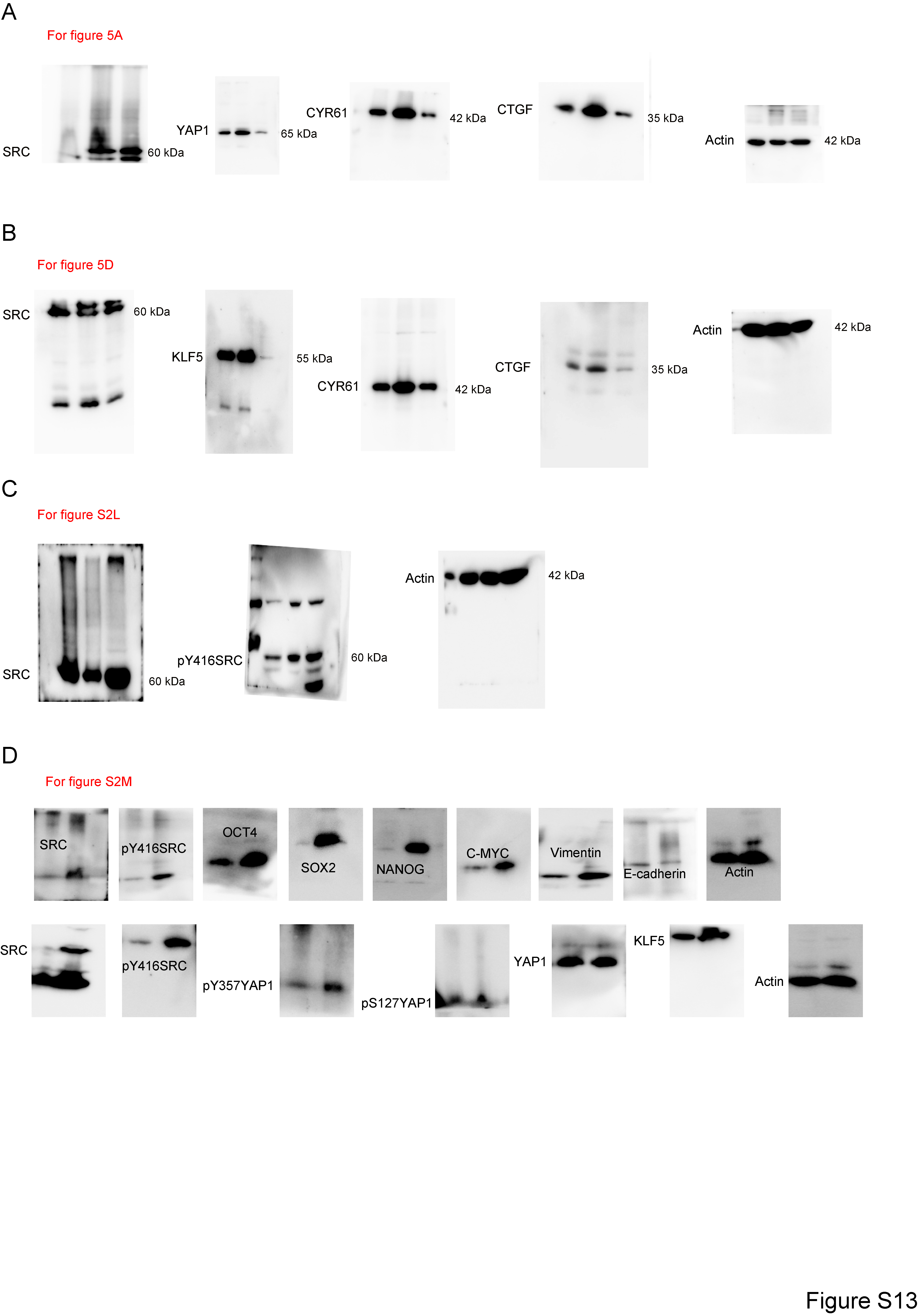

Supplement: Supplementary file 16 — Figure S13. Full western blot images for Figure 5 and S2. Supplementary file16 (TIF 6931 KB) [file 18_2023_4688_MOESM16_ESM.tif]

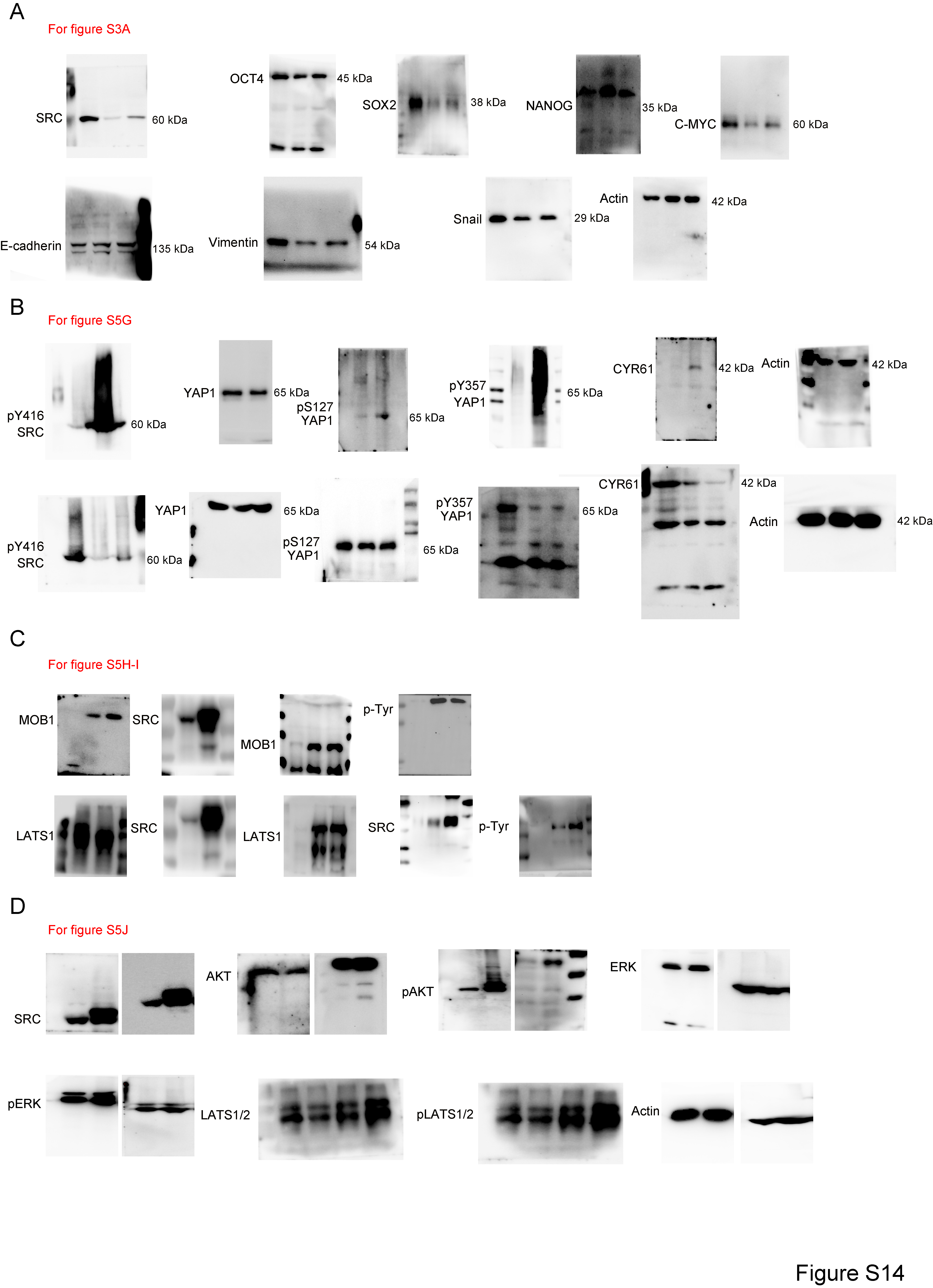

Supplement: Supplementary file 17 — Figure S14. Full western blot images for Figure S3 and S5. Supplementary file17 (TIF 9826 KB) [file 18_2023_4688_MOESM17_ESM.tif]

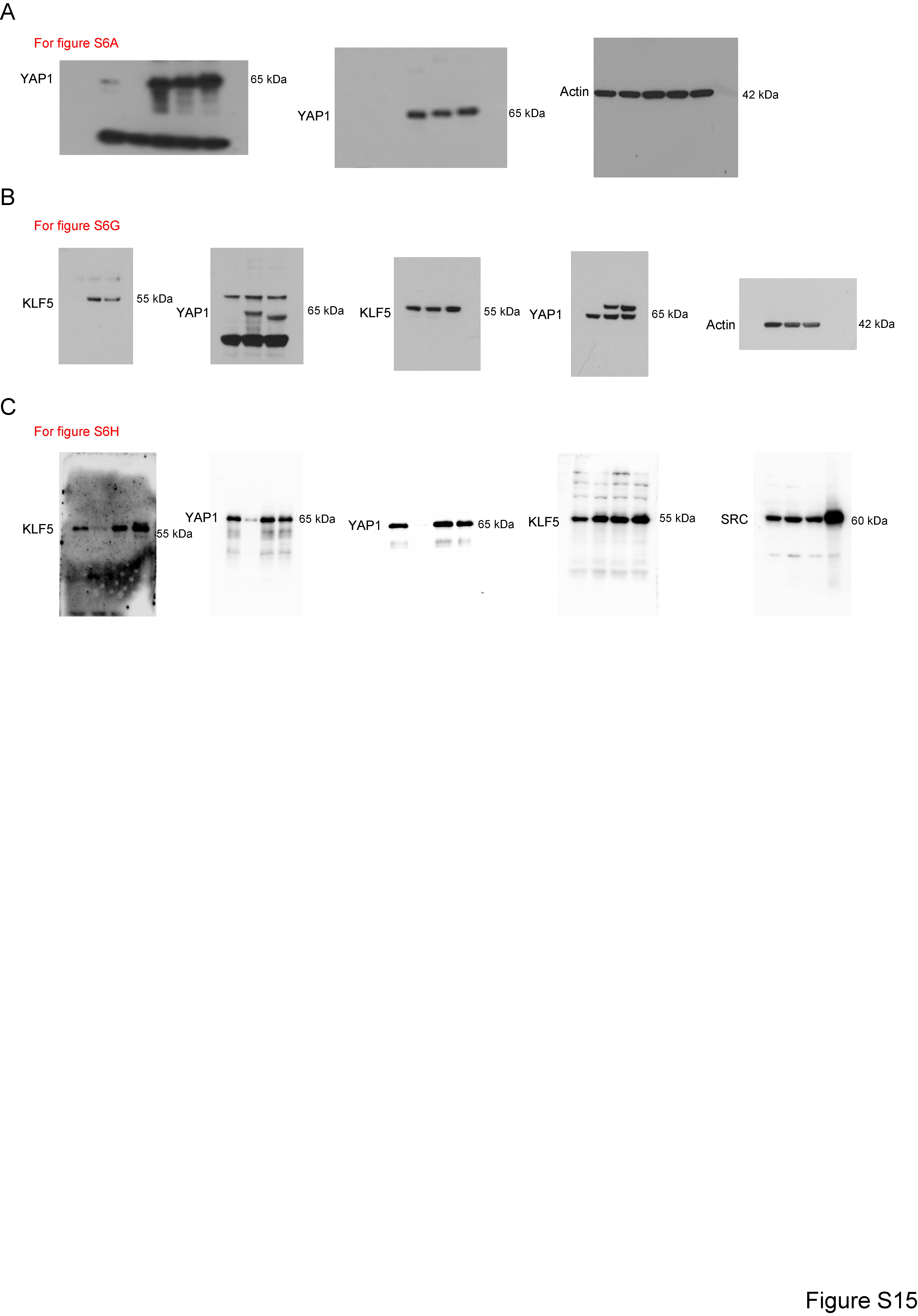

Supplement: Supplementary file 18 — Figure S15. Full western blot images for Figure S6. Supplementary file18 (TIF 4019 KB) [file 18_2023_4688_MOESM18_ESM.tif]
